# Hyperferritinemic sepsis, macrophage activation syndrome, and mortality in a pediatric research network: a causal inference analysis

**DOI:** 10.1186/s13054-023-04628-x

**Published:** 2023-09-06

**Authors:** Zhenziang Fan, Kate F. Kernan, Yidi Qin, Scott Canna, Robert A. Berg, David Wessel, Murray M. Pollack, Kathleen Meert, Mark Hall, Christopher Newth, John C. Lin, Allan Doctor, Tom Shanley, Tim Cornell, Rick E. Harrison, Athena F. Zuppa, Katherine Sward, J. Michael Dean, H. J. Park, Joseph A. Carcillo

**Affiliations:** 1 Department of Computer Sciences, University of Pittsburgh, Pittsburgh, PA USA; 2 Division of Pediatric Critical Care Medicine, Department of Critical Care Medicine, Faculty Pavilion, Children’s Hospital of Pittsburgh, Center for Critical Care Nephrology and Clinical Research Investigation and Systems Modeling of Acute Illness Center, University of Pittsburgh, Suite 2000, 4400 Penn Avenue, Pittsburgh, PA 15421 USA; 3 Department of Human Genetics, Graduate School of Public Health, University of Pittsburgh, Pittsburgh, PA USA; 4 Department of Pediatrics, Children’s Hospital of Pittsburgh, Pittsburgh, PA USA; 5 Department of Anesthesiology, Children’s Hospital of Philadelphia, Philadelphia, PA USA; 6 Division of Critical Care Medicine, Department of Pediatrics, Children’s National Hospital, Washington, DC USA; 7 Division of Critical Care Medicine, Department of Pediatrics, Children’s Hospital of Michigan, Detroit, MI USA; 8 Central Michigan University, Mt Pleasant, MI USA; 9 Division of Critical Care Medicine, Department of Pediatrics, The Research Institute at Nationwide Children’s Hospital Immune Surveillance Laboratory, and Nationwide Children’s Hospital, Columbus, OH USA; 10 Division of Pediatric Critical Care Medicine, Department of Anesthesiology and Pediatrics, Children’s Hospital Los Angeles, Los Angeles, CA USA; 11 Division of Critical Care Medicine, Department of Pediatrics, St. Louis Children’s Hospital, St. Louis, MO USA; 12 Division of Critical Care Medicine, Department of Pediatrics, C. S. Mott Children’s Hospital, Ann Arbor, MI USA; 13 Division of Critical Care Medicine, Department of Pediatrics, Mattel Children’s Hospital at University of California Los Angeles, Los Angeles, CA USA; 14 Department of Pediatrics, University of Utah, Salt Lake City, UT USA

**Keywords:** Severe sepsis, Multiple organ failure, Immunoparalysis, Thrombocytopenia-associated multiple organ failure, Macrophage activation syndrome, Hyperferritinemic sepsis

## Abstract

**Background:**

One of five global deaths are attributable to sepsis. Hyperferritinemic sepsis (> 500 ng/mL) is associated with increased mortality in single-center studies. Our pediatric research network’s objective was to obtain rationale for designing anti-inflammatory clinical trials targeting hyperferritinemic sepsis.

**Methods:**

We assessed differences in 32 cytokines, immune depression (low whole blood ex vivo TNF response to endotoxin) and thrombotic microangiopathy (low ADAMTS13 activity) biomarkers, seven viral DNAemias, and macrophage activation syndrome (MAS) defined by combined hepatobiliary dysfunction and disseminated intravascular coagulation, and mortality in 117 children with hyperferritinemic sepsis (ferritin level > 500 ng/mL) compared to 280 children with sepsis without hyperferritinemia. Causal inference analysis of these 41 variables, MAS, and mortality was performed.

**Results:**

Mortality was increased in children with hyperferritinemic sepsis (27/117, 23% vs 16/280, 5.7%; Odds Ratio = 4.85, 95% CI [2.55–9.60]; z = 4.728; *P*-value < 0.0001). Hyperferritinemic sepsis had higher C-reactive protein, sCD163, IL-22, IL-18, IL-18 binding protein, MIG/CXCL9, IL-1β, IL-6, IL-8, IL-10, IL-17a, IFN-γ, IP10/CXCL10, MCP-1/CCL2, MIP-1α, MIP-1β, TNF, MCP-3, IL-2RA (sCD25), IL-16, M-CSF, and SCF levels; lower ADAMTS13 activity, sFasL, whole blood ex vivo TNF response to endotoxin, and TRAIL levels; more Adenovirus, BK virus, and multiple virus DNAemias; and more MAS (*P*-value < 0.05). Among these variables, only MCP-1/CCL2 (the monocyte chemoattractant protein), MAS, and ferritin levels were directly causally associated with mortality. MCP-1/CCL2 and hyperferritinemia showed direct causal association with depressed ex vivo whole blood TNF response to endotoxin. MCP-1/CCL2 was a mediator of MAS. MCP-1/CCL2 and MAS were mediators of hyperferritinemia.

**Conclusions:**

These findings establish hyperferritinemic sepsis as a high-risk condition characterized by increased cytokinemia, viral DNAemia, thrombotic microangiopathy, immune depression, macrophage activation syndrome, and death. The causal analysis provides rationale for designing anti-inflammatory trials that reduce macrophage activation to improve survival and enhance infection clearance in pediatric hyperferritinemic sepsis.

**Supplementary Information:**

The online version contains supplementary material available at 10.1186/s13054-023-04628-x.

## Introduction

An autopsy audit from 1992 to 2017 estimated that one of five deaths worldwide were attributable to sepsis, with 3 million occurring in children and 8 million occurring in adults [1]. Hyperferritinemia has been associated with increased sepsis mortality in single-center studies [2–8]. Brazilian pediatric investigators were first to report that hyperferritinemic sepsis defined by a ferritin level > 500 ng/ml had the highest mortality [2, 3]. Investigators in Washington State reported incremental increases in need for intensive care and also mortality in hospitalized children with ferritin levels > 1,000 and > 3,000 ng/mL [8]. In adults, Rosario et al. used the term Hyperferritinemic Syndrome to describe this condition in adult patients with macrophage activation syndrome (MAS) related to Still’s disease, septic shock, and catastrophic antiphospholipid with ferritin levels > 3,000 ng/mL [9]. Lachman et al. reported extreme hyperferritinemia (> 9,083 ng / mL) was most commonly seen in adults with septic shock and poor outcomes [10].

Macrophage activation has been linked to sepsis and multiple organ failure in adult autopsies. In three single-center autopsy studies [11–13], investigators reported that hyperplasia of activated, hemophagocytic histiocytes/macrophages was associated with a multiple organ failure pattern characterized by hepatobiliary dysfunction and disseminated intravascular coagulation with increased IL-10, IL-6, IL-1, and IL-8 levels. They correlated this condition to the presence of sepsis and numbers of blood transfusions received. Shakoory et al. performed a *post hoc* analysis of a multicenter trial and reported that a 3-day infusion of the anti-inflammatory IL-1 receptor antagonist protein reduced mortality in a dose-dependent manner in adults with septic shock and features of macrophage activation syndrome including hepatobiliary dysfunction plus disseminated intravascular coagulation, or hyperferritinemia > 2,000 ng/mL [14]. Two multicenter cohorts have corroborated that adults with hyperferritinemic sepsis and the multiple organ failure pattern of combined hepatobiliary dysfunction and disseminated intravascular coagulation have circulating cytokine response profiles reflective of macrophage activation [15–17]. Kernan et al. reported that hyperferritinemic sepsis and MAS can have pathogenic variants related to treatable inherited errors of immunity [18, 19]. There remains a gap in knowledge as to whether therapeutically targeting macrophage activation in personalized clinical trials might benefit patients with hyperferritinemic sepsis and MAS.

To gain important background data, rationale, and impetus for designing and performing anti-inflammatory clinical trials targeting pediatric hyperferritinemic sepsis in our multicenter research network, we herein test the hypothesis that children with hyperferritinemic sepsis (> 500 ng/mL) would demonstrate increased inflammatory cytokinemia and immune depression [20], viral DNAemia [21], thrombotic microangiopathy [22], MAS [14] and death [15–17]. We hypothesized that mortality rates would increase with increasing hyperferritinemia level categories (ferritin 500–999, 1,000–2,999, 3000–9,999, and ≥ 10,000 ng/mL) [2, 3, 8–10]. To gain insights into which modifiable inflammatory cytokines might be optimal therapeutic targets in anti-inflammatory clinical trials designed to improve hyperferritinemic sepsis survival, we applied causal inference analysis to 32 inflammatory cytokines, two biomarkers of immune depression and thrombotic microangiopathy, seven viral DNAemias, five categorical ferritin levels, MAS, and death.

## Materials and methods

The present study analyzes clinical data and bio-banked samples available from children previously enrolled in our nine center *Eunice Kennedy Shriver* National Institutes of Child Health and Human Development Collaborative Pediatric Critical Care Research Network Phenotyping Pediatric Sepsis-induced Multiple Organ Failure (PHENOMS) study [23], approved by the central Institutional Review Board (IRB) of the University of Utah, IRB #70,976. Written informed consent was obtained from one or more parents/guardians for each child. Assent was garnered when the child was able. Patients were enrolled from 2015 to 2017. The details of the clinical protocol have been published [23]. Children qualified for enrollment if they (1) were between the ages of 44 weeks gestation to 18 years; (2) were suspected of having infection and two or more of four systemic inflammatory response criteria [24]; (3) had one or more organ failures defined by modified Proulx et al. criteria [25]; and (4) had an indwelling arterial line or central venous catheter for blood drawing. Children were excluded from enrollment if there was a lack of commitment to aggressive care.

Clinical data were assessed daily until 28 days or discharge from the PICU. These included demographic variables (age, sex, ethnicity, previously healthy status, post-op status), Pediatric Risk of Mortality Score 3 (PRISM-3) to assess severity of illness at admission, and organ failures (Central Nervous System = Glasgow Coma Scale score < 12 not explained by use of sedation; Cardiovascular = requirement for vasoactive agents for systolic blood pressure < 5^th^ percentile for age; Respiratory = PaO2/FiO2 ratio < 300 requiring mechanical ventilation; Renal = oliguria and serum creatinine > 1 mg/dL; Hepatic = ALT > 100 and bilirubin > 1 mg/dL; Hematologic = platelet count < 100 K and INR > 1.5) to assess organ failure index or the number of organ failures. Blood samples were obtained after 24 h of sepsis and twice a week thereafter. The blood samples were analyzed for measurement of ferritin as well as 32 additional cytokine biomarkers, ADAMTS13 activity, whole blood ex vivo TNF response to endotoxin, and seven viral DNAemias.

Thrombocytopenia-associated multiple organ failure (TAMOF) was defined by ADAMTS13 activity < 57% with new onset thrombocytopenia < 100,000, plus acute kidney injury measured by creatinine > 1 mg/dL with oliguria or need for continuous renal replacement therapy or dialysis [26]. Immunoparalysis was defined as whole blood ex vivo TNFα response to endotoxin < 200 pg/mL beyond day three of sepsis [27, 28]. Macrophage activation syndrome was defined by platelet count < 100,000 + INR > 1.5 + ALT > 100 IU/L + bilirubin > 1 mg/dL [14–16, 23]. Death was defined by death in the hospital.

Plasma for cytokine measurement was divided into three assays. IL-18, IL-18BP, and CXCL9 were measured at 25-fold dilution [29]. IFNβ, sCD163, and IL-22 were measured by BioPlex inflammatory flex-set assay per manufacturer’s instructions (Bio-Rad). The remainder were measured by BioPlex Group I/II flex-set assay (Bio-Rad). All cytokines were measured on a BioPlex 200 System (Bio-Rad). The functional assays were measured as previously described. DNA was extracted from frozen plasma samples using the NuclieSENS easyMag automated nucleic acid extractor (bioMerieux) and tested using quantitative real-time polymerase chain reactions (qPCR) for cytomegalovirus (CMV), Epstein-Barr virus (EBV), herpes simplex virus (HSV), human herpesvirus-6 (HHV-6), parvovirus B-19, BK virus, adenovirus, and torque teno virus (TTV) [30].

We compared admission characteristics, outcomes, and 32 cytokines and functional biomarkers across 397 patients in five ferritin groups (< 500, 500–999, 1,000–2,999, 3,000–9,999, and ≥ 10,000 ng/mL), MAS, and mortality groups. We presented continuous variables with median (IQR) or mean (sd) and categorical variables with the percentage value (%). To compare patients visually, we utilized heatmaps with hierarchical clustering as well as bar plots and violin plots to illustrate the characteristics, outcomes, and cytokine differences across the groups. To compare patients statistically, we used Kruskal–Wallis tests for continuous data and the chi-square test for categorical data. Fisher exact tests were applied for groups containing less than five individuals. The threshold for statistical significance was 0.05 for two-sided tests after adjustment for multiple testing. Holm–Bonferroni (BH) correction was applied to correct for multiple testing. Odds ratios were presented with 95% confidence intervals. Adjusted odds ratios for ferritin’s association with mortality were modeled by adding baseline epidemiologic characteristics as well as variables associated with univariate analysis with increasing ferritin and / or death. All analyses were performed with R version 3.6.2.

### Causal inference method

We performed causal inference to observe potential causal associations in the data. With the same set of variables as above as input, we learned causality in a two-step approach. First, associations were identified between variables using a mixed graphical model (MGM) approach coupled with sparsity parameters defined separately for different types of data, named stable edge-specific penalty selection (MGM-StEPS) [31]. Then, we normalized the variables and determined the causal direction of each association using degenerate Gaussian (DG) score (see Additional file 1) [32]. For both the MGM-StEPS algorithm and DG score calculation, we set all parameters to default values. Under fundamental causal assumptions (see Additional file 1), the resulting directed acyclic graph (DAG) allowed us to identify potential causal associations as well as third variables including confounding, mediators, collider bias, and M-bias among the causal associations [33]. By presenting the causal pathways in directed acyclic graph (DAG) [34–36], we could identify not only the causal relationships, but also the third variables that would affect each causal relationship. To facilitate correct interpretations of the potential causal findings, we explicitly reported our findings as “causal association” not “causal effect.” Since our inference was based on observational data that are subject to biases from confounding, selection, and measurement, our inference can result in an underestimate or overestimate of the effect of interest and thus we use the term causal association.

## Results

Of the 401 patients enrolled and reported in the Phenotyping Pediatric Sepsis-induced Multiple Organ Failure study (PHENOMS), 397 had ferritin levels, 32 cytokines, whole blood ex vivo TNF response to endotoxin, and ADAMTS13 activity assayed for this analysis (CONSORT diagram, Additional file 1: eFigure 1). Individual patient ferritin values ranged from < 10 ng/mL to > 100,000 ng/mL with marked skewness. The continuous variable ferritin displays a quadratic relationship with death in Additional file 1: eFigure 2 [2]. The odds ratio of mortality increases as the continuous integer value of ferritin increases within this range of ferritin values (Odds Ratio 1.0000099 [95% CI 1.0000071–1.000013]; *P*-value = 0.00000003808).

**Fig. 1:**
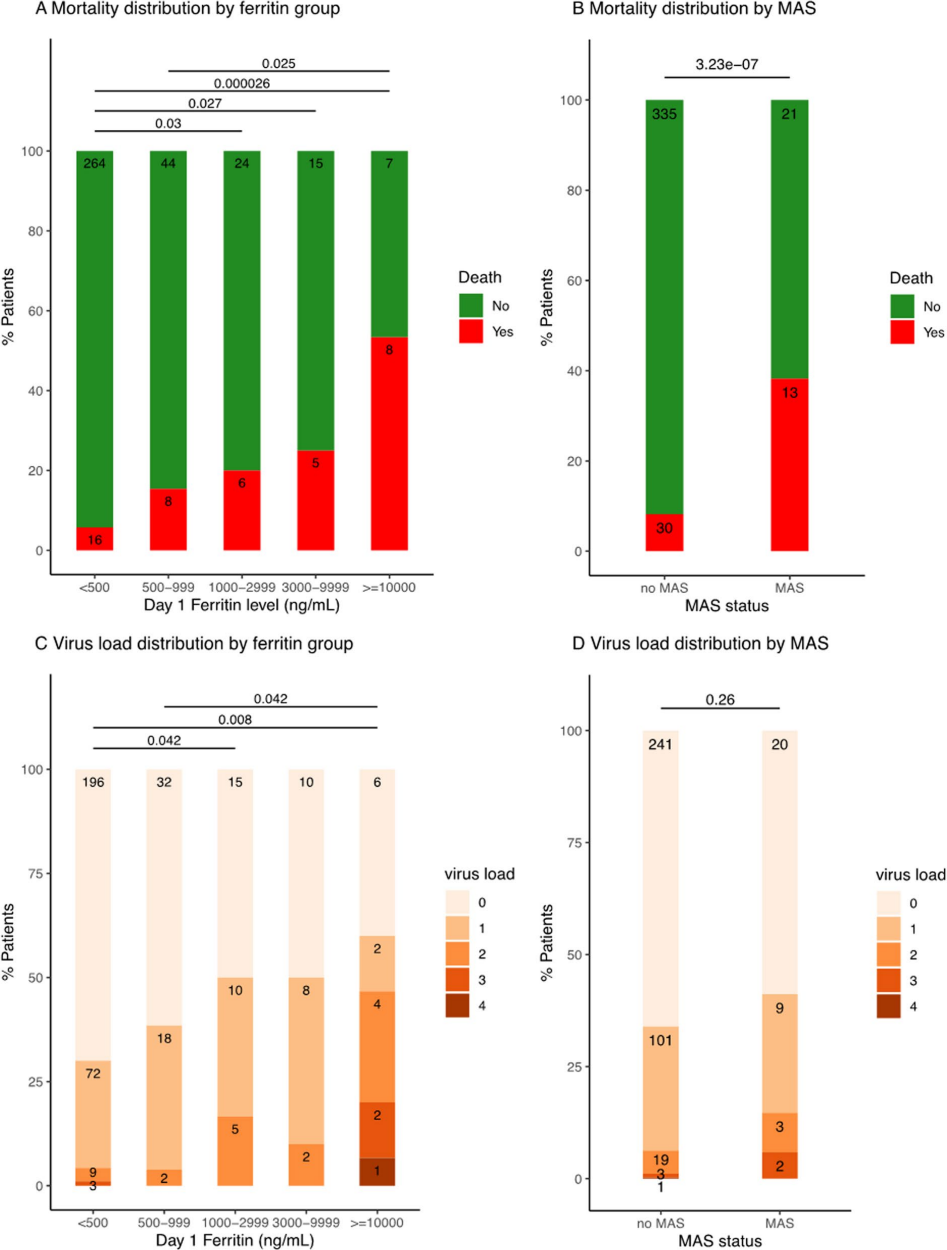
Mortality and virus DNAemia count distribution by ferritin level and MAS category **A** Mortality distribution according to ferritin category; **B** Mortality distribution by MAS (macrophage activation syndrome) category; **C** Number of circulating DNA viruses (count) by ferritin categories; **D** Number of circulating DNA viruses by MAS (macrophage activation syndrome) category

**Fig. 2:**
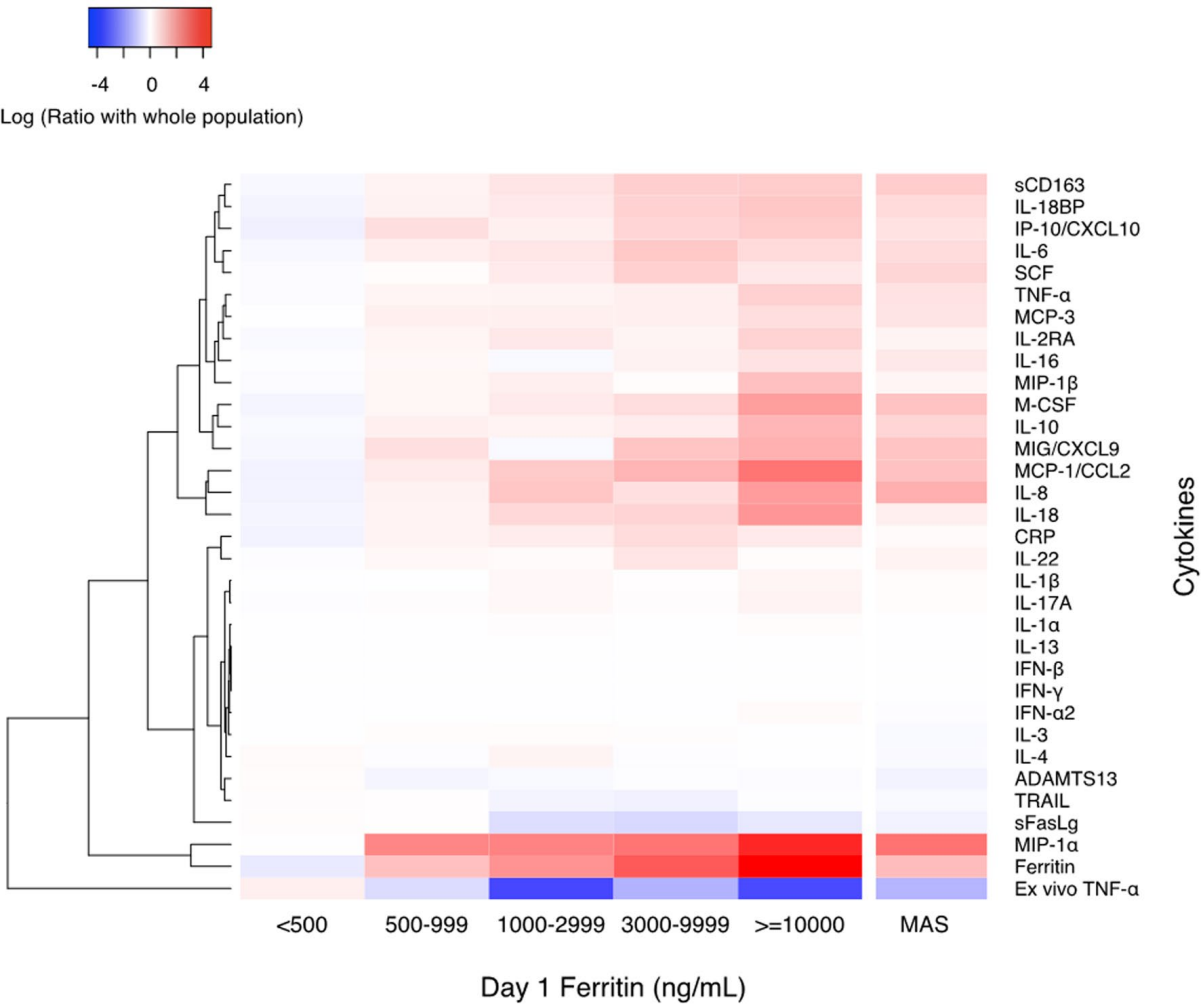
Cytokine heatmap of ferritin categories and MAS—The heatmap shows the log ratio of the median biomarker values for various markers of the host response and their hierarchical cluster relationships. Red represents a greater median biomarker value for that phenotype compared with the median for the entire study cohort, whereas blue represents a lower median biomarker value compared with the median for the entire study cohort

Of these 397 children, 117 (29%) had hyperferritinemia > 500 ng / mL and 280 (71%) did not (Table 1, Fig. 1). Admission characteristics associated with hyperferritinemia > 500 ng/mL included chronic illness, immunocompromise, increased severity of illness, increased number of organ failures, fungal infection, red blood cell transfusion, and platelet transfusion (*P*-value < 0.05) (Table 1). Children with hyperferritinemia > 500 ng / mL had higher levels of C-reactive protein, sCD163, IL-22, IL-18, IL-18 binding protein, MIG/CXCL9, IL-1β, IL-6, IL-8, Il-10, IL-17A, IFN-γ, IP10/CXCL10, MCP-1/CCL2, MIP-1α, MIP-1β, TNF, MCP-3, IL-2RA, IL-16, M-CSF, and SCF; lower ADAMTS13 activity, sFasL levels, whole blood ex vivo TNF response to endotoxin, and TRAIL levels (*P*-value < 0.05) (Fig. 2, Additional file 1: eTable 1, eFigure 3); and more Adenovirus, BK virus, and multiple virus DNAemias (*P*-value < 0.05) (Table 2, Fig. 1). Macrophage activation syndrome developed in 24/117 (21%) of children with hyperferritinemic sepsis and 10/280 (4%) of children with sepsis without hyperferritinemia (Odds Ratio 6.95; 95% CI 3.21–15.12; z = 4.91; *P*-value < 0.0001) (Table 3). Frequency of MAS increased as ferritin levels increased, from 4% (ferritin < 500 ng/mL) to 17% (ferritin 500 to 999 ng/mL), to 17% (ferritin > 1,000 to 2,999 ng/mL), to 10% (ferritin 3,000 to 9,999 ng/mL), and to 53% (ferritin ≥ 10,000 ng/mL) (Table 3).

**Table 1:** Day 1 admission characteristics of ferritin categories and MAS status

Admission Characteristic^a^	Ferritin Level					no MAS	MAS
	< 500	500–999	1000–2999	3000–9999	≥ 10,000		
No. of patients, N (%)	280 (71)	52 (13)	30 (8)	20 (5)	15 (4)	363(91)	34 (9)
Age, median (IQR), y	5.1(1.3,11.7)	4.7(0.5, 13)	5.5(3.8,11.5)	12.2(7.8,14.5)^a^	8.9(6.3–11.2)	5.7(1.5,12.0)	5.6(1.1,13.2)
Sex, N (%)							
Female***	129 (46)	19 (37)	14(47)	8 (40.0)	7 (47)	171(47)	6(18)
Male***	151 (54)	33 (63)	16(53)	12 (60.0)	8 (53)	192(53)	28(82)
Race, N (%)							
White	189 (68)	34 (65)	23(77)	12 (60)	11 (73)	243(67)	26 (77)
Black	60 (21)	10 (19)	6 (20)	3 (15)	2 (13)	77 (21)	4 (12)
Asian	11 (4)	5 (10)	1 (3)	2 (10)	0 (0)	19 (5)	0 (0)
Other	20 (7)	3 (6)	0 (0)	3 (15)	2 (13)	24 (7)	4 (12)
Ethnicity, N (%)							
Non-Hispanic	220 (79)	44 (85)	25 (83)	15 (75)	15 (100)	290 (80)	29 (85)
Hispanic	48 (17)	7 (14)	4 (13)	5 (25.0)	0 (0.0)	60 (17)	4 (12)
Previous healthy, N (%)***	142 (51) ^c,d^	25 (48)^c^	4 (13)	3 (15)	3 (20)	169 (46)	8 (23)*
Immunocompromised, N (%)***	38 (14)	16 (31)^a^	23 (77)^a,b^	14 (70)^a^	11 (73)^a^	85 (23)	17 (50)**
PRISM Score, median (IQR)***	7 (3–13)	10 (6–15)	12 (9–16)	9 (5–15.0)	13 (8–19)	8 (3–14)	13 (10- 19)***
OFI, median (IQR)*	2 (1,2)	2 (1,2)	2 (1,2)	2 (1, 3)	3 (1, 3)	2 (1, 2)	2 (2,4) ***
Bacterial infection, N (%)	94 (34)	23 (44)	10(33)	10 (50)	3 (20)	127(35)	13 (38)
Viral infection, N (%)	88 (31) ^c^	13 (25)	2 (7)	4 (20)	6 (40)	105(29)	8 (23.5)
Co-infection, N (%)	21(8)	4 (8)	0 (0)	2 (10)	3 (20)	27 (7)	3 (9)
Fungal infection, N (%)**	0 (0)	2 (4)	1 (3)	0 (0)	1 (7)	2 (1)	2 (6)*
PRBC Transfusion, N (%)***	42 (15)	14 (27)	19 (63)^a,b^	9 (45)^a^	5 (33)	71 (19)	18 (53)***
Platelet Transfusion, N (%)***	13 (5)	8 (15) ^a^	16 (53) ^a,b^	9 (45)^a^	9 (60) ^a,b^	37 (10)	18 (53)***

Comparisons were performed between the group with ferritin < 500 and ferritin ≥ 500, and the group with MAS and without MAS. Kruskal–Wallis test was used for continuous variables, and the χ2 test or the Fisher’s exact test (group sample size < 10) was used for discrete variables. ***: *P*-value < 0.001 **: *P*-value < 0.01 *: *P*-value < 0.05

*IQR* interquartile range, *PRISM* Pediatric Risk of Mortality Index, *OFI* organ failure index, is an integer score reflecting the number of organ failures. OFI Scores are either 0 or 1 for cardiovascular, hepatic, hematologic, respiratory, neurological, and renal, and summed for total range of 0 to 6. Co-infection = bacteria + virus infections. Immunocompromise – Cancer, transplantation, use of immune suppressant therapies

^a^The outcome characteristic is significantly higher than ferritin < 500 group (*P*-value < 0.05)

^b^The outcome characteristic is significantly higher than ferritin 500–999 group (*P*-value < 0.05)

^c^The outcome characteristic is significantly higher than ferritin 1000–2999 group (*P*-value < 0.05)

^d^The outcome characteristic is significantly higher than ferritin 3000–9999 group (*P*-value < 0.05)

**Table 2:** Viral DNAemia according to ferritin categories and MAS status

Outcome^a^	Ferritin Level					No MAS	MAS
	< 500	500–999	1000–2999	3000–9999	≥ 10,000		
No. of patients, N (%)	280 (71)	52 (13)	30 (8)	20 (5)	15 (4)	363 (91)	34 (9)
EBV,N (%)	14 (5)	5 (9)	0 (0)	1 (5)	1 (7)	19 (5)	2 (6)
CMV, N (%)	19 (7)	3 (6)	7 (23)	1 (5)	4 (27)	32 (9)	2 (6)
HSV1, N (%)	4 (1)	1 (2)	1 (3)	0 (0)	0 (0)	5 (1)	1 (3)
Adeno, N (%)**	11 (4)	6 (12)	3 (10)	3 (15)	1 (7)	22 (6)	2 (6)
BK, N (%)*	7 (3)	0 (0.0)	2 (7)	2 (10)	6 (40)^a,b^	14 (4)	3 (9)
Parvo, N (%)	13 (5)	2 (4)	1 (3)	1 (5)	2 (13)	18 (5)	1 (3)
HHV-6, N (%)	31 (11)	5 (10)	6 (20)	4 (20)	6 (40)	42 (12)	10 (29)*
No. of viruses							
Mean (sd)***	0.35(0.60)	0.42(0.57)	0.67(.76)^a^	0.6(0.68)	1.33 (1.35)^a,b^	0.42 (.66)	0.62 (0.88)
Median (IQR)***	0 (0, 1)	0 (0, 1)	0.5 (0,1)^a^	0.5 (0,1)	1(0,2)^a,b^	0 (0,1)	0 (0,1)

Comparisons were performed between the group with ferritin < 500 and ferritin ≥ 500, and the group with MAS and without MAS. Kruskal–Wallis test was used for continuous variables, and the χ2 test or the Fisher’s exact test (group sample size < 10) was used for discrete variables. ***: *P*-value < 0.001 **: *P*-value < 0.01 *: *P*-value < 0.05

^a^The outcome characteristic is significantly higher than ferritin < 500 group (*P*-value < 0.05)

^b^The outcome characteristic is significantly higher than ferritin 500–999 group (*P*-value < 0.05)

^c^The outcome characteristic is significantly higher than ferritin 1000–2999 group (*P*-value < 0.05)

**Table 3:** Outcomes according to ferritin category and MAS status

Outcome	Ferritin level					No MAS	MAS
	< 500	500–999	1000–2999	3000–9999	≥ 10,000		
No. of patients, N (%)	280 (71)	52 (13)	30 (8)	20 (5)	15 (4)	363 (91)	34 (9)
Lymphopenia, N (%)***	140 (50)^c^	25 (48)	5 (17)	5 (25)	4 (27)	162 (45)	17 (50)
MechVent, N (%)**	252 (90)^c^	42 (81)	20 (67)	18 (90)	10 (67)	311 (86)	31 (91)
ECMO, N (%)***	8 (3)	4 (8)	1 (3)	0 (0)	2 (13)	9 (3)	6 (18)***
CRRT, N (%)***	5 (2)	5 (10)^a^	4 (13)	1 (5)^a^	1 (7)^a^	10 (3)	6 (18)***
PLEX, N (%)***	1 (0.3)	1 (2)	1 (3)	0 (0)	2 (13)^a^	2 (0.6)	3 (9)**
Mortality, N (%)***	16 (6)	8 (15)	6 (20)^a^	5 (25)^a^	8 (53)^a,b^	30 (8)	13 (38)***
IPMOF, N (%)***	41 (15)	16 (31)	8 (27)	10 (50)^a^	9 (60)^a^	64 (18)	20 (59)***
TAMOF, N (%)***	12 (4)	9 (17)^a^	5 (17)	4 (20)^a^	7 (47)^a^	16 (4)	21 (62)***
MAS, N (%)***	10 (4)	9 (17)^a^	5 (17)	2 (10)	8 (53)^a^	–	–

Lymphopenia = Absolute Lymphocyte Count < 1,000/mm^3^

Comparisons were performed between the group with ferritin < 500 and ferritin ≥ 500, and the group with MAS and without MAS. Kruskal–Wallis test was used for continuous variables, and the χ2 test or the Fisher’s exact test (group sample size < 10) was used for discrete variables. ***: *P*-value < 0.001 **: *P*-value < 0.01 *: *P*-value < 0.05

*IQR* interquartile range, *MechVent* Mechanical Ventilation, *ECMO* Extracorporeal Membrane Oxygenation, *CRRT* Continuous Renal Replacement Therapies, *PLEX* Plasma Exchange, *IPMOF* immunoparalysis-associated multiple organ failure, *TAMOF* thrombocytopenia-associated multiple organ failure, *MAS* macrophage activation syndrome

^a^The outcome characteristic is significantly higher than ferritin < 500 group (*P*-value < 0.05)

^b^The outcome characteristic is significantly higher than ferritin 500–999 group (*P*-value < 0.05)

^c^The outcome characteristic is significantly higher than ferritin 1000–2999 group (*P*-value < 0.05)

Mortality occurred in 27/117 (23%) of children with hyperferritinemic sepsis > 500 ng/mL compared to 16/280 (6%) of children with sepsis without hyperferritinemia (Odds Ratio 4.85; 95% CI [2.55–9.60]; z = 4.728; *P*-value < 0.0001) (Table 3, Fig. 1). Mortality increased as ferritin category levels increased from 5.7% (ferritin < 500 ng/mL) to 15.4% (ferritin 500 to 999 ng/mL), to 20% (ferritin > 1,000 to < 2,999 ng/mL), to 25% (ferritin > 3,000 to 9,999 ng/mL), and to 53.3% (ferritin ≥ 10,000 ng/mL) (Table 3, Fig. 1). After adjustment for epidemiologic factors including age, sex, race, and ethnicity and admission variables associated with ferritin levels (Table 1) and / or death including previously healthy status, PRISM score, preexisting immunocompromise status, maximum organ failures, presence of fungal infection, or receipt of packed red blood cell transfusion and/or platelet transfusion (Additional file 1: eTables 2–4), these ferritin category levels remained independently associated with increasing mortality odds in a stepwise manner (adjusted odds ratio of mortality 1.044, 95% CI: [1.012–1.078], *P*-value = 0.006483 for each increase in ferritin category level; Additional file 1: eTable 5). Children with hyperferritinemic sepsis also developed more immunoparalysis, TAMOF, and requirement for extracorporeal support (*P*-value < 0.05) but less lymphopenia (Table 3).

Admission characteristics associated with MAS included male sex, chronic illness, immunocompromise, increased severity of illness, increased number of organ failures, fungal infection, red blood cell transfusion, and platelet transfusion (*P*-value < 0.05) (Table 1). Children who developed MAS showed increased ferritin, sCD163, IL-22, IL-18, IL-18 BP, MIG/CXCL9, IL-1β, IL-6, IL-8, IL-10, MCP-1/CCL2, MIP-1α, MIP-1β, TNF, IL-16, M-CSF, and SCF; decreased ADAMTS13 activity, whole blood ex vivo TNF response to endotoxin, and IFN-β (*P*-value < 0.05) (Fig. 2, Additional file 1: eTable 6, eFigure 4); and more Human Herpes Virus 6 DNAemias (*P*-value < 0.05) (Table 2). Mortality occurred in 13/34 (38.2%) of children with MAS compared to 30/363 (8.3%) of children without (Odds Ratio 6.87; 95% CI [3.13–15.08]; z = 4.81; *P*-value < 0.0001) (Fig. 1, Table 3). Children with MAS also developed more immunoparalysis, TAMOF, and requirement for extracorporeal support (*P*-value < 0.05) (Table 3).

**Fig. 3:**
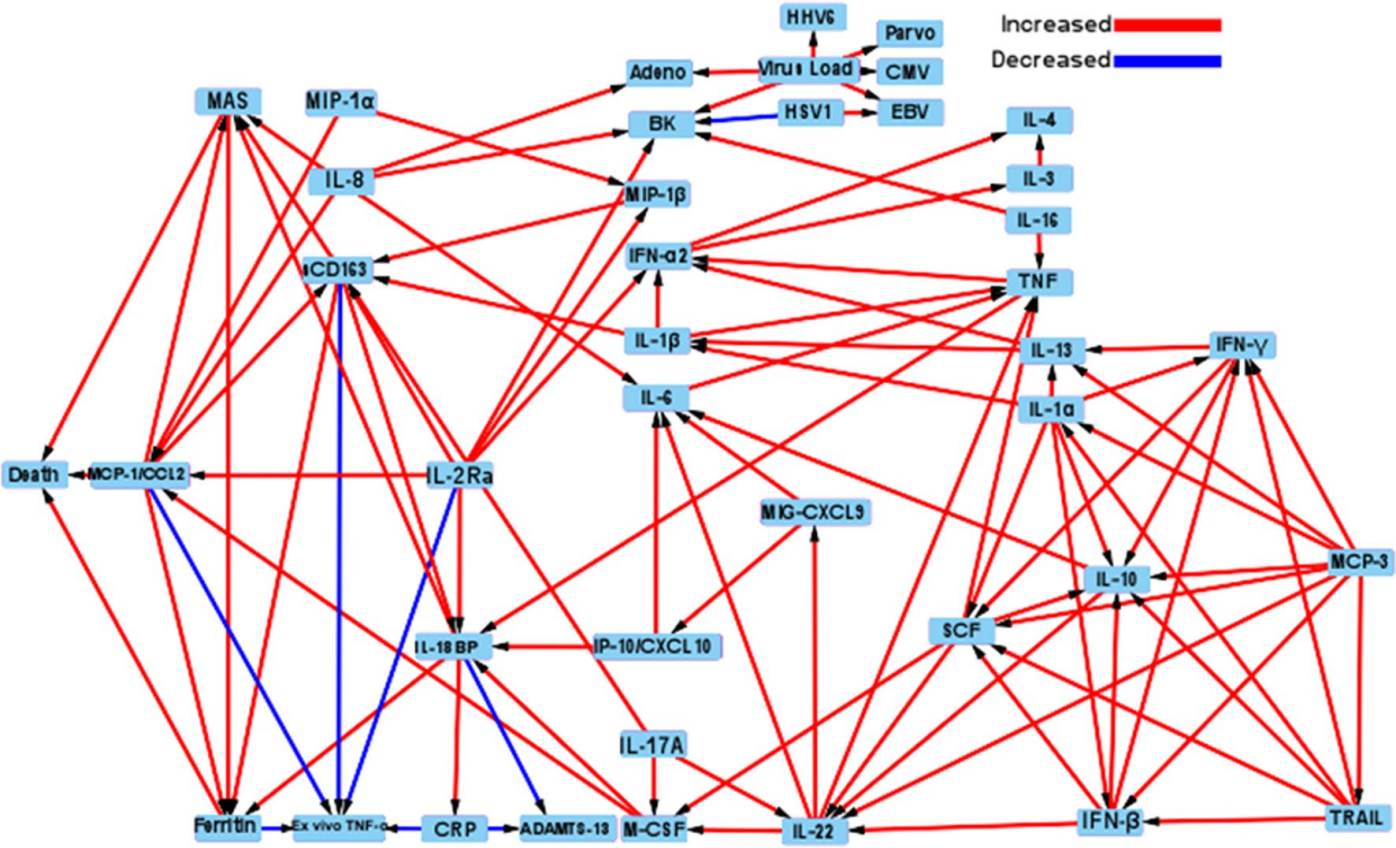
Full causal pathway directed acyclic graph analysis of all causal associations found among cytokines, viral DNAemia, hyperferritinemia, MAS, and death. Causal analysis revealed the associations among all variables/outcomes. Red arrows denote a positive causal association (increased variable/outcome: increased variable/outcome), and blue arrows denote a negative causal association (increased variable/outcome: decreased variable outcome). Arrows denote direction of causality

**Fig. 4:**
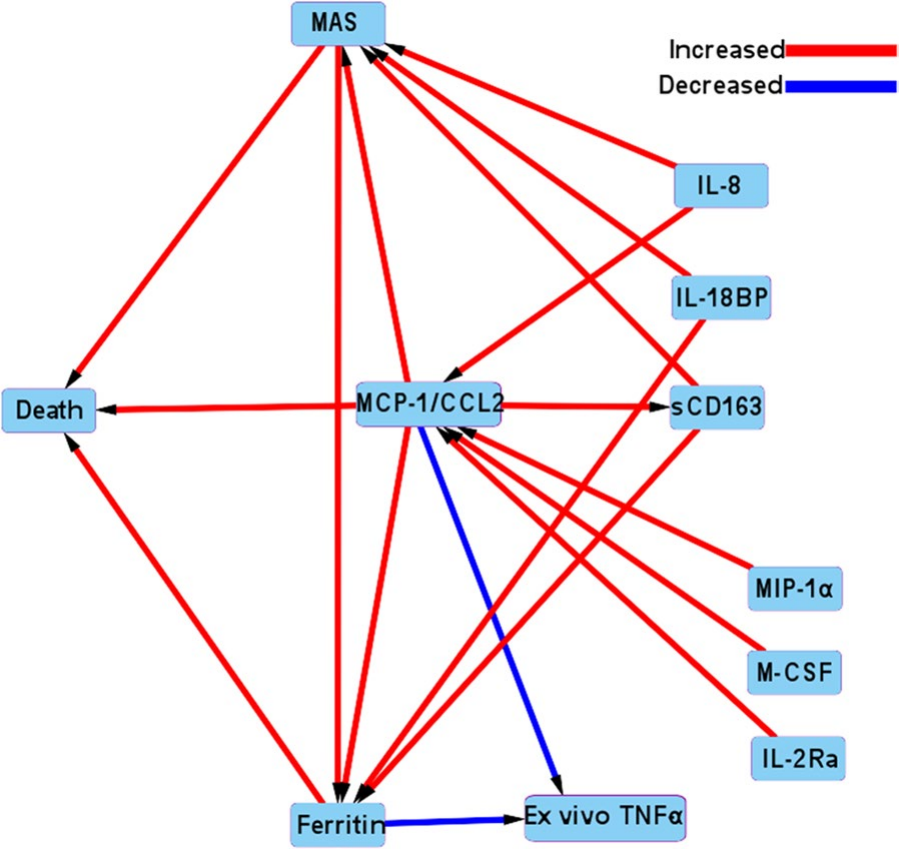
Abridged causal pathway directed acyclic graph analysis of eight cytokines with MAS, hyperferritinemia, and death. Causal analysis revealed the associations among variables/ outcomes. Red arrows denote a positive causal association (increased variable/outcome: increased variable/outcome), and blue arrows denote a negative causal association (increased variable/outcome: decreased variable outcome). Arrows denote direction of causality

Figure 3 shows the pediatric sepsis causal pathway DAG analysis demonstrating all MGM-derived causal associations. Among the 41 identified causal nodes, only increased MCP-1/CCL2, MAS, and hyperferritinemia levels were directly causally associated with death (Figs. 3 and 4, Additional file 1: Table S7). Increased MCP-1/CCL2 levels were also causally associated with increased MAS, hyperferritinemia, and sCD163 levels but decreased whole blood ex vivo TNF response to endotoxin. Macrophage activation syndrome was also causally associated with hyperferritinemia levels, while hyperferritinemia levels were also causally associated with decreased whole blood ex vivo TNF response to endotoxin. Confounders, mediators, and colliders are identified among MCP-1/CCL2, MAS, and hyperferritinemia, with death in Additional file 1: Table S7.

Increased IL-8, IL-18BP, and sCD163 levels were causally associated with MAS; increased IL-8, MIP-1 alpha, M-CSF, and sCD25/IL-2Ra levels were causally associated with increased MCP-1/CCL2 levels; and increased IL-18BP and sCD163 levels were causally associated with hyperferritinemia (Figs. 3 and 4). All identified causal associations are depicted in Fig. 3 to allow inference of potential cytokine modulation strategies.

Whole blood ex vivo TNF response was lower in children with preexisting immunocompromised status (*P* = e2.9e-07) (Additional file 1: eFigure 5) as well as in children with bacterial infection (*P* < 0.05) (Additional file 1: eFigure 6) but higher in children with viral infection (*P* < 0.05) (Additional file 1: eFigure 6). Causal associations of increasing hyperferritinemia category and MCP-1 / CCL2 levels with death and reduced whole blood ex vivo TNF response remained with the addition of preexisting immunocompromise status to the DAG.

## Discussion

Our multicenter study establishes pediatric hyperferritinemic sepsis as a common high-risk condition characterized by hypercytokinemia, viral DNAemia, thrombotic microangiopathy, immune depression, MAS, and death [37]. Nearly, one of three children in our sepsis cohort had ferritin levels > 500 ng/mL. Nearly, two of three children who required continuous renal replacement therapy, one of two children who required extracorporeal membrane oxygenation, three of four who developed MAS, and two of three children who died had ferritin levels > 500 ng/mL. Death increased in a stepwise manner with increasing category levels of hyperferritinemia [8]. Consistent with adult studies, children with hyperferritinemic sepsis had a hypercytokinemia profile reflective of macrophage activation without a commensurate Th2 response (low IL-4 and IL-13) [15–17]. Decreased ADAMTS13 activity with TAMOF reflective of thrombotic microangiopathy [22] and increased circulating IL-10 reduced whole blood ex vivo TNF response to endotoxin, and immunoparalysis reflective of immune depression with reduced ability to kill infection [27, 28, 37] was also observed with increasing ferritin levels [20].

We applied causal analysis to gain inference into potential anti-inflammatory strategies that might reduce inflammation and mortality in these children. Monocyte chemoattractant protein 1 (MCP-1)/CCL 2, MAS, and hyperferritinemia were identified as the only three variables directly causally associated with death. Hyperferritinemia and MCP-1/CCL2 were also causally associated with reduced ex vivo whole blood TNF response to endotoxin implying that targeting hyperferritinemic sepsis and macrophage activation could also improve infection clearance.

Clinical drugs specifically targeting ferritin-related ferritinophagy and ferroptosis such as dexrazoxane and dexmedetomidine have benefit in experimental sepsis models [38–40]. Similarly, the selective MCP-1/CCL2 inhibitor Bindarit is beneficial in experimental sepsis [41]. However, visualization of the causal pathways with third factor analysis in the DAG implies that therapies which target MCP-1/CCL2, MAS, and hyperferritinemia all together will be more efficacious than therapies that target any one of these alone. Demirkol previously reported that the combined therapies of methylprednisolone, IVIG, and plasma exchange therapy reversed pediatric hyperferritinemic sepsis and MAS, improved outcome, and facilitated infection clearance in the low middle-income PICU setting [42]. Methylprednisolone and IVIG are anti-inflammatory therapies commonly used to treat MAS [43, 44], while plasma exchange has been shown to remove M-CSF, IL-8, and ferritin [44]. Our DAG analysis shows M-CSF and IL-8 were causally associated with MCP-1/CCL2.

Our DAG analysis further identified causal associations between IL-18BP, sCD163, M-CSF, IL-8, MIP-1 alpha, and IL-2Ra/sCD25 with increased MCP-1/CCL2, MAS, and hyperferritinemia. This provides rationale for testing combinations of specific inhibitors and/or signaling pathway inhibitors with activity against many or all of these cytokines. For practical reasons, our network has chosen interleukin 1 receptor antagonist protein (Anakinra) for our first placebo controlled clinical trial. It is already FDA approved for use in neonatal onset multisystem inflammatory disease (NOMID) with a proven excellent safety profile. This biologic blocks MCP-1/CCL2 production during experimental radiation injury [45] reduces sCD163, IL-2RA/sCD25, and ferritin production in COVID19 patients [46, 47], inhibits IL-8 and MIP-1 alpha production in mixed lymphocyte cultures without affecting t-cell function [48], and reverses MAS in children [49]. Interleukin 1β was causally associated with TNF and sCD163 in our DAG. In turn, TNF had causal association with IL-18BP which had causal association with MAS and hyperferritinemia. Similarly, sCD163 had causal association with hyperferritinemia and MAS. From this, we infer that IL-1β inhibition by Anakinra might decrease hyperferritinemia and MAS. Our research network is presently enrolling children in the targeted reversal of inflammation in pediatric sepsis-induced MODS trial (TRIPS: NCT05267821) testing the ability of seven days of Anakinra to improve 28-day MODS resolution in hyperferritinemic sepsis [50].

The major limitation of our study is that the numbers of hyperferritinemia / MAS / mortality patients are small. We are unable to fully evaluate the role of preexisting immunocompromised status. Even though we found increasing ferritin category levels remained associated with mortality after controlling for preexisting immunocompromised conditions and increasing ferritin categories and MCP-1/CCL2 levels remained causally associated with death and low whole blood ex vivo TNF response after adding preexisting immunocompromise status, only five of our 401 patients had hematopoietic stem cell transplantation which is the severest form of immunocompromise. Concern about generalizability of our findings will remain pending further investigations in independent pediatric cohorts.

Because our study was designed to corroborate previous findings of single-center pediatric and multicenter adult studies in our independent multicenter pediatric cohort, the biomarkers and ferritin category levels were pre-selected as a confirmatory list rather than an agnostic one. Specimen collections occurred after 24 h of severe sepsis giving time for ferritin levels to reach their peak; however, cytokines expected to peak in the first 6 h of sepsis such as IL-1β and TNF may have been higher if sampled earlier. Causal relationships might differ at different time points. Further, observations that specific cytokines show causal associations with mortality do not mean that their blockade will improve survival. Rather these findings are hypothesis generating. Clinical trials such as TRIPS are needed to confirm proposed in vivo effects. Findings in pediatric hyperferritinemic sepsis-induced MODS are not generalizable to all critical illness as hyperferritinemia is less predictive of poor outcomes in adult community-acquired pneumonia unaccompanied by hematologic cancers [51–54]. Findings are also expected to differ in resource-poor settings where malnutrition leads to lower baseline ferritin levels, and tropical infections including dengue virus, hemorrhagic fevers, severe malarial anemia, Ebola virus, Leishmaniasis, and scrub typhus lead to higher ferritin levels during sepsis [55–63]. Whereas hyperferritinemia is linked to mortality in COVID19 patients [64], it is important to emphasize that our cohort is pre-COVID19 pandemic.

## Conclusions

This multicenter investigation establishes hyperferritinemic sepsis as a high-risk hyperinflammatory condition in children. The causal inference pathway analysis provides rationale for designing clinical trials testing anti-inflammatory therapies that modulate reticuloendothelial system activation to improve outcome and enhance infection clearance in children with hyperferritinemic sepsis.

### Supplementary Information

**Additional file 1**. Supplemental Digital Content.

## Data Availability

All data generated or analyzed during this study are included in this published article and its Additional file 1. The datasets used and /or analyzed during the current study are available from the corresponding author on reasonable request. The PHENOMS database can also be uploaded from the NICHD sponsored DASH website.

## Abbreviations

MOF: Multiple organ failure
PICU: Pediatric intensive care unit
IPMOF: Immunoparalysis MOF
TAMOF: Thrombocytopenia-associated MOF
MAS: Macrophage activation syndrome
SMOF: Sequential MOF
IVIG: Intravenous immune globulin
NIGMS: National Institutes of General Medical Sciences
PHENOMS: PHENOtyping pediatric sepsis-induced Mof Study
ADAMTS13: A disintegrin and metalloproteinase with a thrombospondin type 1 motif, member 13
PRISM: Pediatric RISk of Mortality
SIRS: Systemic Inflammatory Response Syndrome
CRRT: Continuous renal replacement therapy
ECMO: Extracorporeal membrane oxygenator
NICHD: National Institutes of Child Health and Human Development
DAG: Directed acyclic graph
MGM: Mixed graphical model
StEPS: Stable edge-specific penalty selection
