# Supplementary material for: Hyperferritinemic sepsis, macrophage activation syndrome, and mortality in a pediatric research network: a causal inference analysis
Source: Crit Care. 2023 Sep 6;27:347. doi: 10.1186/s13054-023-04628-x (PMC10481565; doi:10.1186/s13054-023-04628-x)

**Supplemental digital content eMethods, eTable 1-7, eFigures 1-6**

eMethods. Detailed description of causal inference method with references

eTable 1. Biomarkers measured at Day 1 by ferritin categories

eTable 2. Adjusted odds ratio modeling of ferritin category and mortality using patient epidemiology characteristics with and without PRISM score

eTable 3. Adjusted odds ratio modeling of ferritin category and mortality using patient epidemiology characteristics and PRISM score with and without pre-existing immunocompromised status

eTable 4. Univariate associations between mortality and the variables other than PRISM that were associated with ferritin categories (eg. previous healthy status, pre-existing immunocompromise status, maximum organ failures, fungal infection, and packed RBC or platelet transfusions).

eTable 5. Adjusted odds ratio modeling of ferritin category and mortality controlling for epidemiology characteristics, PRISM score, Previously Healthy status, pre-existing Immunocompromised status, organ failures, fungal infection, and packed RBC and platelet transfusion

eTable 6. Biomarkers measured at Day 1 by MAS category

eTable 7.  Effects and third variable confounders, mediators, and colliders for the three identified causal associations with death (see Figure 4)

eFigure 1. CONSORT diagram

eFigure 2. Continuous ferritin variable relationship with death

eFigure 3. Cytokine value distribution by ferritin category status

eFigure 4. Cytokine value distribution by MAS status

eFigure 5. Whole blood ex vivo TNF response to endotoxin in children with pre-existing immunocompromised status compared to children without

eFigure 6. Whole blood ex vivo TNF response to endotoxin in children with bacterial infection compared to those without; and, in children with viral infection compared to those without

**Detailed description of causal inference method with references**

In this study, we sought to identify potentially druggable targets in sepsis trials by using a two-stage approach consisting of mixed graphical model (MGM) and degenerate gaussian (DG) model. MGMs have provided simplicity in modeling and the interpretability of results.^1-4^ Thus, they offer a clear benefit over other machine learning methods such as support vector machines (SVMs), random forests (RFs) without loss in classification accuracy.^1^ Several studies also showed that MGMs have theoretical guarantees that their solutions (dependencies and orientations in the graph) are asymptotically correct. Due to these advantages, MGMs have been used as 'causal' graphs in biomedical research^5,6^ with three fundamental causal assumptions: Markov, faithfulness, and sufficiency. The causal Markov condition states that causal relationships among the set of variables in their probability distributions (e.g., Bayesian network) are conditionally independent of their non-descendants given their parents.^7^ The causal faithfulness condition states that all independence relations in the data are consequences of the causal Markov condition. The causal sufficiency condition states that input data measured all the common causes of the measured variables, including confounders. For example, our analysis identified that MAS and MCP-1/CCL2 were confounders of the causal association from ferritin to death; however, no latent (unobserved) confounder is assumed to exist.

Due to MGMs being taken as ‘causal’ graphs, building MGMs on clinical data provides a particular benefit for elucidating clinical effect. That is, under the assumption of no latent confounders, one can select the most informative variables for any outcome of interest based on the graph due to the Markov blanket property. Since the Markov blanket consists of its parent nodes, descendant nodes and other parent nodes of the descendants in the graph, this property makes the target variable effectively independent of any other variables in the data set.

Among various MGMs, MGM-StEPs is an established algorithm that has successfully learned associations in mixed data types from breast cancer patients in TCGA, from the Lung Genomics Resource Consortium (LGRC) cohort, and Pittsburgh Specialized Center of Clinically Oriented Research (SCCOR) cohort.^1^ MGM-StEPs regularizes edges differently by type: continuous-to-continuous, continuous-to-discrete, and discrete-to-discrete. To avoid overfitting, MGM-StEPs uses subsampling to build the most stable graph across the subsamples.

In the second step, we used DG to determine the causal directions in each of the associations identified in the MGM step. DG extends the established causal inference score metric Bayesian information criterion (BIC). BIC was first introduced as an approximation for the marginal likelihood that penalizes the number of instances in the data.^8^ Later it was extended to a more general class of models (including linear Gaussian DAG).^9^ To infer causality from observational data, BIC was proven to be decomposable, score equivalent, and consistent.^10^ A score is decomposable if it decomposes to each node in the graph. In other words, a decomposable score computes the score by estimating the causality in each node in the graph and summarizing it. A score is score equivalent if it gives the same score to any two DAGs that are Markov equivalent. A score is consistent if a model whose parameter space contains the true distribution is ranked better than a model whose parameter space does not. Also, a consistent score ranks better the simpler of two models whose parameter spaces both contain the true distribution. DG extends all three properties for learning directed acyclic graphs (DAGs) from high-dimensional data with mixed data-types and demonstrates a near-perfect performance under certain simulation scenarios of high-dimensional data of mixed types.^11^

In accordance with “Control of Confounding and Reporting of Results in Causal Inference Studies”, we have provided third variables, such as confounders, mediators, and colliders (eTable 7) Confounders are associated with the exposure of interest, a cause of the outcome of interest, and do not reside in the causal pathway between the exposure and outcome. Mediator refers to the variable that is influenced by the exposure, which in turn influences the outcome. A collider is a variable that is caused by at least two other variables (the causing variables “collide” in the collider). An M-bias factor is a special case in which the collider has no causal association with exposure or outcome but is indirectly associated with both through ancestors (causes) of exposure and disease.^12^

1) Sedgewick AJ , Shi I , Donovan RM , et al . Learning mixed graphical models with separate sparsity parameters and stability-based model selection. BMC Bioinformatics 2016;17.doi:10.1186/s12859-016-1039-0 Google Scholar

2) Kitsios GD , Fitch A , Manatakis DV , et al . Respiratory microbiome profiling for etiologic diagnosis of pneumonia in mechanically ventilated patients. Front Microbiol 2018;9.doi:10.3389/fmicb.2018.01413 Google Scholar

3) Manatakis DV , Raghu VK , Benos PV . piMGM: incorporating multi-source priors in mixed graphical models for learning disease networks. Bioinformatics 2018;34:i848–56.doi:10.1093/bioinformatics/bty591 Google Scholar

4) Abecasis I , Sedgewick AJ , Romkes M , et al . PARP1 rs1805407 increases sensitivity to PARP1 inhibitors in cancer cells suggesting an improved therapeutic strategy. Sci Rep 2019;13.Google Scholar

5) Spirtes P , Glymour CN , Causation SR . Causation, prediction, and search. Adaptive computation and machine learning. Cambridge Mass: MIT Press, 2000: 543.Google Scholar

6) Pearl J . Causality: models, reasoning, and inference. Cambridge UK: Cambridge University Press, 2009.Google Scholar

7) H. E. Kyburg, “Probabilistic Reasoning in Intelligent Systems: Networks of Plausible Inference by Judea Pearl,” Journal of Philosophy, vol. 88, no. 8. Morgan Kaufmann, pp. 434–437, 1991, doi: 10.5840/jphil199188844.

8) Gideon Schwarz et al. Estimating the dimension of a model. The annals of statistics, 6: 461–464, 1978.

9) Dominique Haughton. On the choice of a model to fit data from an exponential family. The Annals of Statistics, 16:342–355, 1988.

10) David Maxwell Chickering. Optimal structure identification with greedy search. Journal of machine learning research, 3:507–554, 2002.

11) <https://doi.org/10.1093/nar/gkaa350>

12) Lederer DJ, Bell SC, Branson RD, et al. Control of Confounding and Reporting of Results in Causal Inference Studies. Guidance for Authors from Editors of Respiratory, Sleep, and Critical Care Journals. Ann Am Thorac Soc. 2019 Jan;16(1):22-28. doi: 10.1513/AnnalsATS.201808-564PS. Erratum in: Ann Am Thorac Soc. 2019 Feb;16(2):283. PMID: 30230362.

**eTable 1. Biomarkers measured at Day 1 by ferritin categories**

| **Cytokines** | **Ferritin Level** | | | | |
| --- | --- | --- | --- | --- | --- |
|  | **<500** | **500-999** | **1000-2999** | **3000-9999** | **>=10000** |
| CRP *** | 8.03 (2.71, 14.84) | 12.13 (6.85, 21.86)^a^ | 13.69 (5.16, 19.18) | 18.32 (8.91, 23.61)^a^ | 14.39 (8.45, 21.91) |
| Ferritin *** | 148.60 (80.07, 227.57) | 664.10 (596.50, 791.68)^a^ | 1495.95 (1257.00, 2418.25) ^a,b^ | 4184.90 (3618.68, 5797.25) ^a,b,c^ | 21990.00 (14139.00, 52872.00) ^a,b,c,d^ |
| ADAMTS13 *** | 75.00 (58.00, 92.25) ^b^ | 60.00 (46.75, 77.25) | 64.50 (57.00, 76.25) | 68.50 (50.00, 84.00) | 67.00 (50.00, 85.50) |
| sFasLg *** | 47.54 (32.24, 79.17) ^c^ | 45.31 (31.79, 70.53) | 25.00 (15.07, 48.11) | 22.94 (18.49, 77.12) | 30.28 (17.58, 57.07) |
| Ex vivo TNF-α *** | 559.72 (245.02, 1049.22) ^b,c,d,e^ | 227.88 (45.34, 508.08) ^c,e^ | 15.58 (6.87, 480.60) | 109.88 (22.48, 451.27) | 16.50 (2.37, 92.44) |
| sCD163 *** | 262253.80 (174321.50, 400867.20) | 359168.75 (221941.80, 593974.28) ^a^ | 464860.40 (322521.48, 865142.48) ^a,b^ | 694781.10 (396293.65, 1343913.72) ^a,b^ | 729494.40 (302637.35, 1198137.20) ^a,b^ |
| IFN-β | 6.40 (6.40, 8.20) | 6.40 (6.40, 6.40) | 6.40 (6.40, 12.50) | 6.40 (6.40, 21.50) | 6.40 (6.40, 6.40) |
| IL-22 *** | 24.80 (20.10, 31.90) | 29.50 (20.10, 36.60) | 28.30 (23.60, 37.72) | 41.30 (23.33, 87.88) ^a^ | 27.10 (22.40, 70.90) |
| IL-18 *** | 357.25 (218.50, 557.82) | 521.50 (350.52, 764.28) ^a^ | 858.20 (513.35, 1173.55) ^a,b^ | 915.05 (605.78, 1588.28) ^a,b^ | 2781.90 (808.15, 3916.25) ^a,b^ |
| IL-18BP *** | 13390.70 (7426.60, 23564.15) | 20102.95 (13569.88, 31872.05) ^a^ | 23787.60 (13814.33, 36268.88) ^a,b^ | 35545.20 (25748.88, 52019.62) ^a,b,c^ | 43580.90 (25117.10, 69127.05) ^a,b,c^ |
| MIG/CXCL9 *** | 700.00 (388.40, 1399.85) | 1402.35 (587.48, 3994.20) ^a^ | 733.60 (488.60, 3013.90) | 2282.30 (656.32, 8278.12) ^a^ | 3262.20 (1943.40, 32045.95) ^a,c^ |
| IL-1β * | 2.80 (2.10, 3.20) | 2.80 (2.40, 3.20) | 3.20 (2.52, 3.65) | 2.85 (2.32, 3.20) | 3.30 (2.60, 3.90) |
| IL-4 | 5.10 (3.50, 6.80) | 4.50 (3.50, 6.50) | 5.70 (3.50, 7.32) | 4.50 (3.50, 6.08) | 4.70 (3.50, 5.85) |
| IL-6 *** | 7.80 (6.00, 14.30) | 11.85 (7.87, 28.70) ^a^ | 13.80 (9.32, 26.35) ^a^ | 23.50 (6.65, 47.65) ^a^ | 16.70 (10.85, 38.20) ^a^ |
| IL-8 *** | 44.50 (29.70, 76.80) | 67.95 (43.67, 154.72) ^a^ | 152.00 (77.60, 353.12) ^a,b^ | 94.25 (57.30, 286.67) ^a^ | 322.00 (98.80, 4224.95) ^a,b,d^ |
| IL-10 *** | 20.50 (16.30, 27.50) | 29.30 (20.12, 43.02) ^a^ | 27.80 (24.15, 50.08) ^a^ | 31.20 (23.25, 80.52) ^a^ | 83.90 (34.60, 209.50) ^a,b,c^ |
| IL-13 | 3.10 (3.10, 3.90) | 3.10 (3.10, 3.90) | 3.10 (3.10, 4.82) | 3.10 (3.10, 3.72) | 3.10 (3.10, 3.70) |
| IL-17A ** | 18.30 (15.60, 22.60) | 19.55 (16.50, 26.80) | 21.30 (17.62, 24.08) | 19.55 (17.17, 23.65) | 23.40 (18.25, 26.80) |
| IFN-γ * | 2.80 (2.80, 3.00) | 2.80 (2.80, 2.85) | 2.80 (2.80, 3.80) | 2.80 (2.80, 4.78) | 2.80 (2.80, 3.40) |
| IP-10/CXCL10 *** | 570.20 (309.40, 1545.20) | 1315.30 (416.53, 3766.62) ^a^ | 962.80 (359.47, 1379.32) | 1572.05 (740.90, 5927.20) ^a^ | 1840.10 (1135.05, 7969.40) ^a,c^ |
| MCP-1/CCL2 *** | 113.80 (54.60, 247.70) | 202.95 (124.23, 517.10) ^a^ | 372.80 (134.90, 893.38) ^a^ | 539.20 (107.95, 1642.80) ^a^ | 1722.30 (774.05, 3511.60) ^a,b,c^ |
| MIP-1α *** | 0.60 (0.60, 4.50) | 5.30 (0.60, 13.37) ^a^ | 5.55 (0.60, 14.57) ^a^ | 7.20 (1.35, 29.00) ^a^ | 29.40 (8.50, 49.40) ^a,b,c^ |
| MIP-1β *** | 42.80 (29.30, 61.40) | 53.85 (40.40, 76.65) ^a^ | 60.75 (35.90, 126.55) ^a^ | 48.95 (32.55, 101.90) | 142.50 (52.45, 222.15) ^a,b^ |
| TNF-α *** | 70.90 (52.30, 94.80) | 88.80 (67.95, 119.80) ^a^ | 90.00 (56.15, 141.55) | 97.70 (61.63, 208.82) ^a^ | 171.20 (108.20, 220.50) ^a,b,c^ |
| MCP-3 * | 92.40 (92.40, 166.00) | 119.50 (92.40, 166.00) | 119.50 (92.40, 176.95) | 119.50 (92.40, 172.78) | 166.00 (92.40, 209.35) |
| IFN-α2 | 125.70 (105.80, 140.20) | 125.70 (105.80, 136.82) | 124.30 (110.72, 154.80) | 125.70 (111.58, 136.82) | 135.70 (116.75, 144.40) |
| IL-1α | 9.40 (9.40, 13.20) | 9.40 (9.40, 13.20) | 9.65 (9.40, 24.65) | 9.40 (9.40, 9.90) | 9.90 (9.40, 22.40) |
| IL-2RA *** | 343.50 (213.90, 539.20) | 449.35 (284.53, 696.68) | 566.80 (307.78, 958.02) ^a^ | 456.00 (352.30, 821.15) ^a^ | 821.90 (261.15, 1095.20) |
| IL-3 | 612.20 (496.10, 724.40) | 636.60 (520.78, 734.32) | 648.25 (529.00, 739.48) | 624.40 (477.90, 656.85) | 612.20 (543.90, 682.20) |
| IL-16 *** | 554.20 (398.10, 711.10) | 655.55 (439.12, 888.10) ^a^ | 513.95 (353.30, 908.28) | 717.50 (487.20, 1196.80) ^a^ | 939.60 (688.55, 1659.05) ^a,b,c^ |
| M-CSF *** | 25.60 (15.60, 41.70) | 35.60 (25.60, 86.95) ^a^ | 44.10 (25.60, 82.60) ^a^ | 55.75 (37.33, 107.85) ^a^ | 172.80 (88.85, 509.15) ^a,b,c,d^ |
| SCF *** | 148.90 (114.80, 215.30) | 167.50 (106.70, 274.40) | 233.15 (157.85, 302.75) ^a^ | 368.90 (150.85, 525.18) ^a,b^ | 240.40 (181.10, 415.30) ^a^ |
| TRAIL * | 37.90 (30.40, 55.40) | 37.25 (28.80, 52.00) | 30.40 (24.43, 46.02) | 28.50 (22.90, 46.93) | 35.40 (25.40, 50.40) |

CRP = mg/dL; Ferritin = ng/mL; ADAMTS 13 activity = % of control; all other variables = pg/mL

Comparisons were performed between the group with ferritin < 500 and ferritin >= 500. Kruskal-Wallis test was used for continuous variables. ***: p-value < 0.001 **: p-value < 0.01 *: p-value < 0.05.

a. The outcome characteristic of this computable phenotype is significantly higher than ferritin < 500 group (p-value < 0.05)

b. The outcome characteristic is significantly higher than ferritin 500-999 group (p-value < 0.05)

c. The outcome characteristic is significantly higher than ferritin 1000-2999 group (p-value < 0.05)

d. The outcome characteristic is significantly higher than ferritin 3000-9999 group (p-value < 0.05)

**eTable 2. Adjusted odds ratio modeling of ferritin category and epidemiologic factors with mortality including and not including PRISM score.**

**Univariable Model with PRISM score Model without PRISM Score**

**Adj OR [95% CI]; p-value Adj OR [95% CI]; p-value**

**Ferritin Category** **1.086 [1.056, 1.116]; 7.906e-9 1.091 [1.061, 1.121] 1.235e-9**

**Sex** 1.035 [0.977, 1.097]; 2.460e-1 1.036 [0.977, 1.099] 2.339e-1

**Age** 1.000 [0.999, 1.000]; 4.982e-1 1.000 [1.000, 1.000] 2.788e-1

**Ethnicity**

**Not Hispanic / Latino** 1.048 [0.965, 1.138]; 2.690e-1 1.061 [0.977, 1.153] 1.600e-1

**Unknown** 0.946 [0.797, 1.124]; 5.295e-1 0.966 [0.813, 1.148] 6.972e-1

**Race**

**Black** 1.019 [0.879, 1.181]; 8.061e-1 1.020 [0.879, 1.184] 7.933e-1

**White**  0.987 [0.860, 1.132]; 8.481e-1 0.993 [0.865, 1.140] 9.163e-1

**Unknown** 1.110 [0.934, 1.320]; 2.365e-1 1.102 [0.926, 1.311] 2.764e-1

**PRISM score** **1.005 [1.001, 1.009]; 7.175e-3**

**eTable 3. Adjusted odds ratio modeling of ferritin category with mortality including patient epidemiology characteristics and PRISM score with and without pre-existing immunocompromised status.**

**Univariable Model without Model with Pre-existing**

**Immunocompromised Status**

**Adj OR [95% CI]; p-value Adj OR [95% CI]; p-value**

**Ferritin category 1.086 [1.056, 1.116]; 7.906e-9 1.083 [1.051, 1.117]; 4.769e-7**

**Sex** 1.035 [0.977, 1.097]; 2.460e-1 1.035 [0.976, 1.097]; 2.479e-1

**Age** 1.000 [1.000, 1.000]; 4.982e-1 1.000 [1.000, 1.000]; 5.264e-1

**Ethnicity**

**Not Hispanic / Latino** 1.048 [0.965, 1.138]; 2.690e-1 1.048 [0.965, 1.139]; 2.640e-1

**Unknown** 0.946 [0.797, 1.124]; 5.295e-1 0.946 [0.797, 1.124]; 5.319e-1

**Race**

**Black** 1.019 [0.879, 1.181]; 8.061e-1 1.019 [0.879, 1.181]; 8.069e-1

**White** 0.987 [0.860, 1.132]; 8.481e-1 0.987 [0.860, 1.132]; 8.513e-1

**Unknown** 1.110 [0.934, 1.320]; 2.365e-1 1.112 [0.935, 1.322]; 2.310e-1

**PRISM score 1.005 [1.001, 1.009]; 7.175e-3**  **1.005 [1.001, 1.009]; 7.425e-3**

**Immunocompromised** 1.011 [0.938, 1.090]; 7.690e-1

**eTable 4. Univariate associations of mortality the six variables other than PRISM that were associated with ferritin categories in Table 1 (eg. previous healthy status, pre-existing immunocompromise status, maximum organ failures, fungal infection, and packed RBC or platelet transfusions).**

**Variable Mortality OR [95% CI] p-value**

**Previously Healthy 0.903 [0.850, 0.959] 1.016e-3**

**Immunocompromised 1.124 [1.049, 1.203] 9.826e-4**

**OFI 1.092 [1.056, 1.130] 4.764e-7**

**Fungal infection 1.913 [1.418, 2.581] 2.714e-5**

**PRBC transfusion 1.268 [1.183, 1.359] 7.318e-11**

**Platelet transfusion 1.374 (1.265, 1.493) 3.526e-13**

**eTable 5. Adjusted odds ratio modeling of ferritin category with mortality controlling for epidemiology characteristics and PRISM score with and without the variables Previously Healthy status, Immuncompromised status, organ failures (OFI), fungal infection, packed RBC transfusion and platelet transfusion.**

**Univariables Model without other variables Model with other variables**

**Odds Ratio [95% CI]; p-value Odds Ratio [95% CI]; p-value**

**Ferritin category 1.086 [1.056, 1.116]; 7.906e-9 1.044 [1.012, 1.078]; 6.483e-3**

**Sex** 1.035 [0.977, 1.097]; 2.460e-1 1.029 [0.974, 1.088]; 3.120e-1

**Age** 1.000 [0.999, 1.000]; 4.982e-1 1.000 [1.000, 1.000]; 2.492e-1

**Ethnicity**

**Not Hispanic/Latino** 1.048 [0.965, 1.138]; 2.690e-1 1.048 [0.969, 1.134]; 2.428e-1

**Unknown** 0.946 [0.797, 1.124]; 5.295e-1 0.946 [0.803, 1.114]; 5.049e-1

**Race**

**Black** 1.019 [0.879, 1.181]; 8.061e-1 1.014 [0.882, 1.166]; 8.455e-1

**White** 0.987 [0.860, 1.132]; 8.481e-1 0.980 [0.861, 1.116]; 7.632e-1

**Unknown** 1.110 [0.934, 1.320]; 2.365e-1 1.093 [0.928, 1.288]; 2.860e-1

**PRISM score 1.005 [1.001, 1.009]; 7.175e-3** 1.002 [0.998, 1.006]; 2.976e-1

**Previously Healthy** 0.970 [0.912, 1.031]; 3.303e-1

**Immunocompromise** 0.954 [0.884, 1.030]; 2.320e-1

**OFI** 1.022 [0.986, 1.059]; 2.424e-1

**Fungal infection 1.589 [1.201, 2.102]; 1.273e-3**

**PRBC transfusion 1.140 [1.060, 1.226]; 4.699e-4**

**Platelet transfusion 1.174 [1.064, 1.296]; 1.563e-3**

**eTable 6. Biomarkers measured at Day 1 by MAS category**

| **Cytokines** | **no MAS** | **MAS** |
| --- | --- | --- |
| CRP | 9.82 (3.22, 17.08) | 10.67 (3.69, 20.37) |
| Ferritin | 197.00 (94.05, 513.50) | 720.20 (365.15, 5864.50)*** |
| ADAMTS13 | 72.00 (57.00, 89.00) | 57.50 (42.25, 70.25)** |
| sFasLg | 46.03 (29.40, 72.93) | 36.23 (23.85, 78.76) |
| Ex vivo TNF-α | 442.36 (119.96, 1044.88) | 112.62 (16.61, 753.44)** |
| sCD163 | 278169.80 (184736.10, 438995.70) | 718199.30 (356088.60, 1262555.50)*** |
| IFN-β | 6.40 (6.40, 8.20) | 6.40 (6.40, 6.40)* |
| IL-22 | 24.80 (20.10, 34.20) | 31.90 (24.80, 53.10)** |
| IL-18 | 408.90 (249.17, 698.10) | 564.80 (402.90, 1623.80)** |
| IL-18BP | 14989.10 (8698.30, 27279.60) | 30224.20 (19510.30, 42572.90)*** |
| MIG/CXCL9 | 753.60 (418.65, 1817.05) | 2321.30 (662.60, 4205.90)*** |
| IL-1β | 2.80 (2.10, 3.20) | 3.00 (2.60, 3.30)* |
| IL-4 | 4.70 (3.50, 6.80) | 4.30 (3.50, 6.00) |
| IL-6 | 8.40 (6.20, 18.20) | 16.50 (11.40, 38.10)*** |
| IL-8 | 51.00 (31.40, 92.20) | 229.60 (84.70, 541.00)*** |
| IL-10 | 21.70 (17.20, 32.00) | 47.30 (30.50, 93.20)*** |
| IL-13 | 3.10 (3.10, 4.30) | 3.10 (3.10, 3.40) |
| IL-17A | 19.10 (16.50, 23.40) | 20.00 (17.40, 25.90) |
| IFN-γ | 2.80 (2.80, 3.00) | 2.80 (2.80, 3.80) |
| IP-10/CXCL10 | 711.50 (334.20, 1897.00) | 1236.80 (480.20, 3196.70) |
| MCP-1/CCL2 | 132.80 (67.80, 323.00) | 426.50 (190.90, 1722.30)*** |
| MIP-1α | 0.60 (0.60, 6.60) | 7.40 (5.00, 17.50)*** |
| MIP-1β | 44.80 (30.20, 67.10) | 54.10 (42.50, 90.70)** |
| TNF-α | 74.90 (55.40, 101.80) | 122.00 (69.60, 190.30)*** |
| MCP-3 | 92.40 (92.40, 166.00) | 147.80 (92.40, 180.60) |
| IFN-α2 | 125.70 (105.80, 140.20) | 120.00 (105.80, 140.20) |
| IL-1α | 9.40 (9.40, 13.20) | 9.40 (9.40, 19.40) |
| IL-2RA | 371.80 (235.80, 597.50) | 457.60 (300.70, 962.50) |
| IL-3 | 636.60 (529.00, 724.40) | 558.80 (496.10, 659.90) |
| IL-16 | 557.30 (405.90, 743.30) | 858.00 (585.10, 1234.00)*** |
| M-CSF | 28.10 (16.30, 47.10) | 88.90 (57.60, 171.80)*** |
| SCF | 152.90 (115.40, 228.50) | 334.00 (212.40, 609.80)*** |
| TRAIL | 36.60 (29.10, 54.20) | 32.90 (25.40, 46.60) |

CRP = mg/dL; Ferritin = ng/mL; ADAMTS 13 activity = % of control; all other variables = pg/mL

Comparisons were performed between the group with and without MAS. Kruskal-Wallis test was used for continuous variables. ***: p-value < 0.001 **: p-value < 0.01 *: p-value < 0.05.

**eTable 7 – Effects and third variable confounders, mediators, and colliders for the three identified causal associations with death (see Figure 3 and 4)**

| **Cause** | **Effect** | **Direction** | **Confounder** | **Mediator** | **Collider** |
| --- | --- | --- | --- | --- | --- |
| Ferritin | Death | Positive | MAS  MCP1/CCL2 |  |  |
| Ferritin | Ex vivo TNF response | Negative |  |  |  |
|  |  |  |  |  |  |
| MAS | Death | Positive | MCP1/CCL2 | Ferritin |  |
| MAS | Ferritin | Positive | IL-18BP  MCP1/CCL2  sCD163 |  | Death |
|  |  |  |  |  |  |
| MCP1/CCL2 | Death | Positive |  | Ferritin  MAS |  |
| MCP1/CCL2 | Ferritin | Positive |  | MAS | Death |
| MCP1/CCL2 | MAS | Positive |  |  | Death  Ferritin |

**eFigure 1. CONSORT diagram**


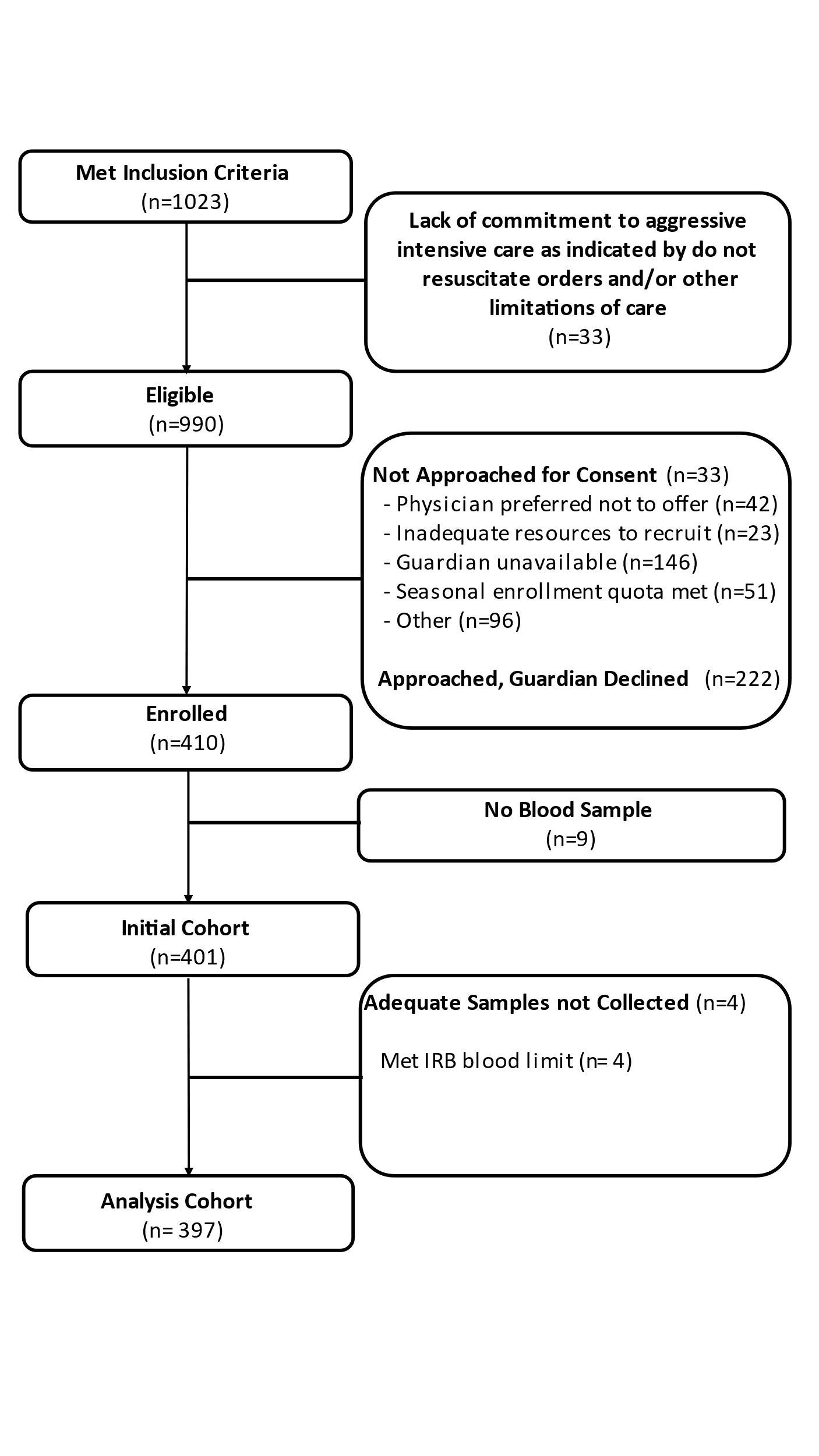


**eFigure 2 Relationship of continuous ferritin variables to death**

**
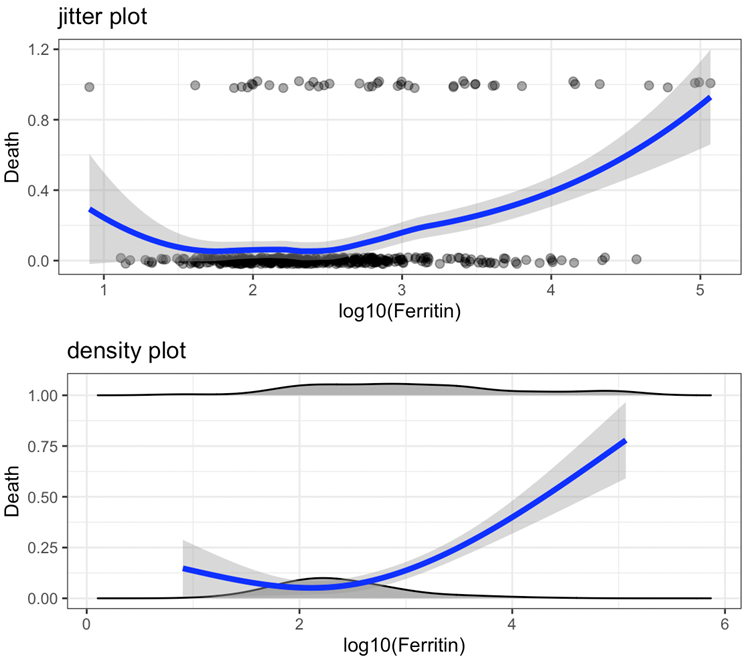
**

To visualize the relationship between ferritin as a continuous variable with mortality, we first generate a jitter plot. In this plot, y-axis represents death status of patients and dots around 0 or 1 indicate death or survival respectively. The x-axis represents ferritin level after log10 transformation given the skewed distribution of the raw value. The blue curve was generated based on smooth method “loess” which does not assume the distribution by plotting a non-parametric line. Then we use a density plot to visualize the data. In this plot, density of death and survival were generated above the 0 and 1 line and the blue curve was generated based on smooth method “glm”, family “binomial”, and formula “y ~ splines::ns(x, c(2)) ”. In this case, we can model the relationship with a parametric approach to enable a more reasonable confidence interval. From both plots, we can observe a quadratic relationship between log10(ferritin) and mortality with a knot around 2 (i.e. ferritin equals 100) consistent with the previous report of Garcia et al [2] showing highest mortality with hyperferritinemia (ferritin levels > 500 ng / mL).

**eFigure 3. Cytokine value distribution by ferritin category status**

**
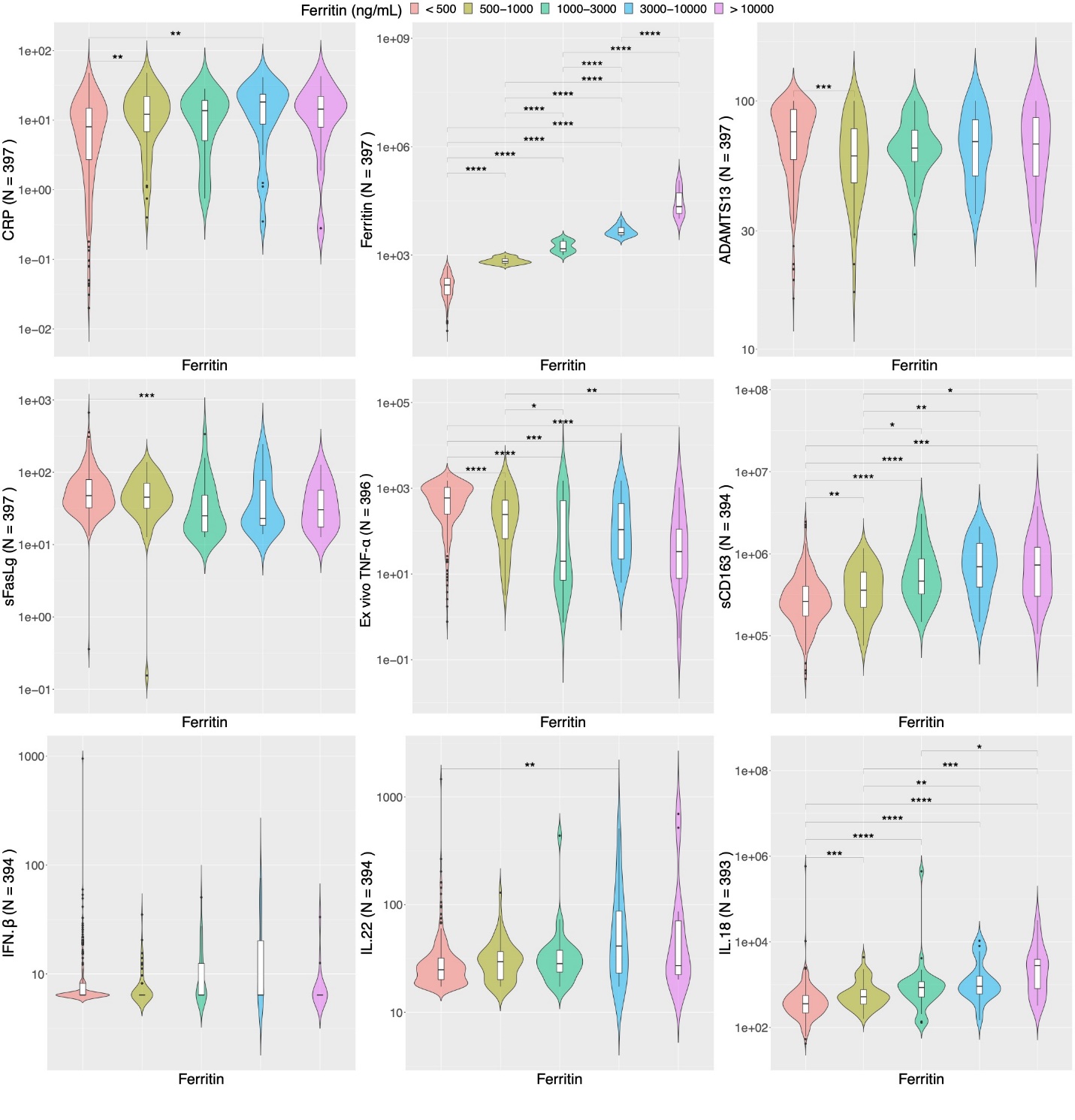
**


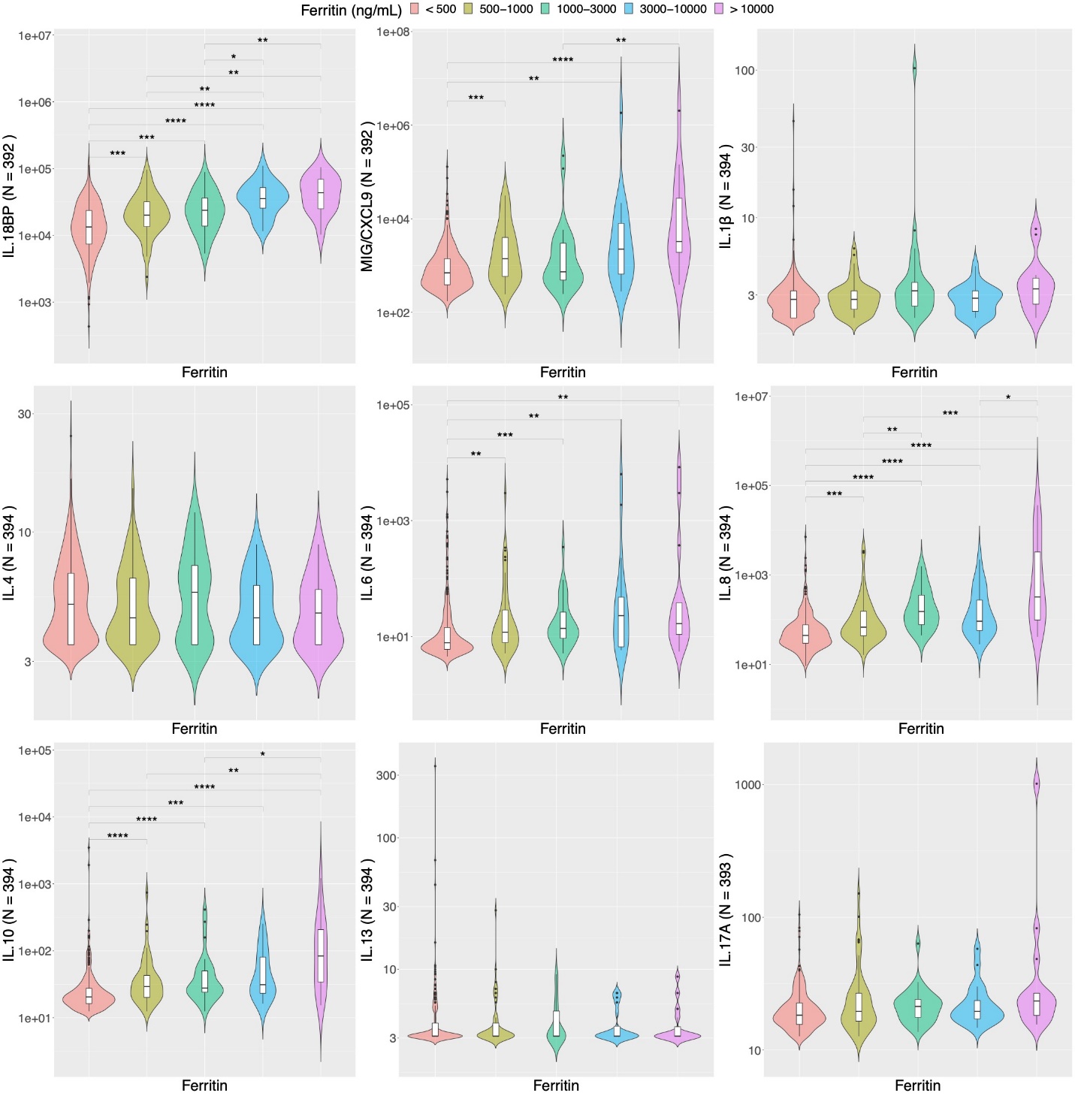


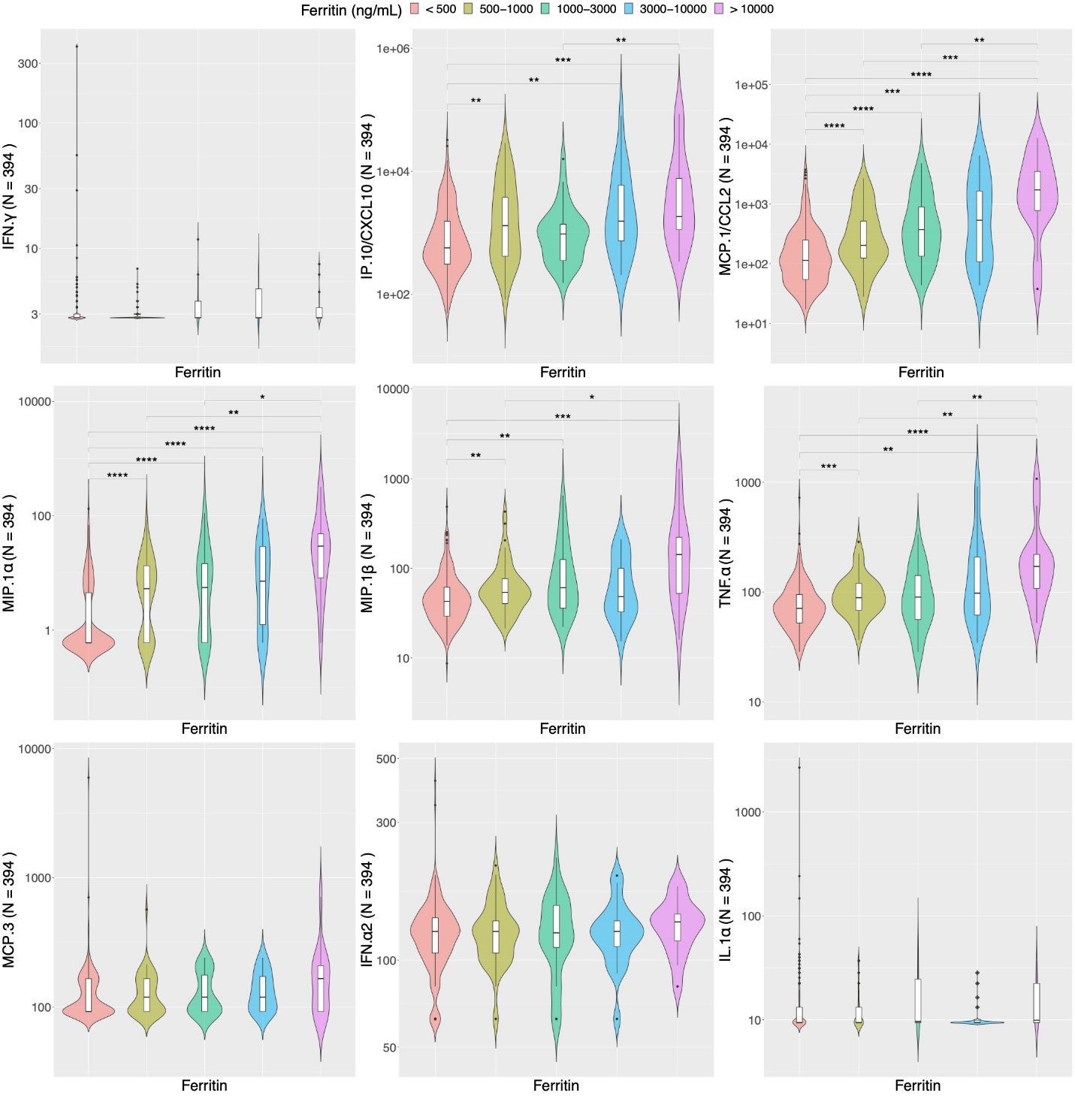


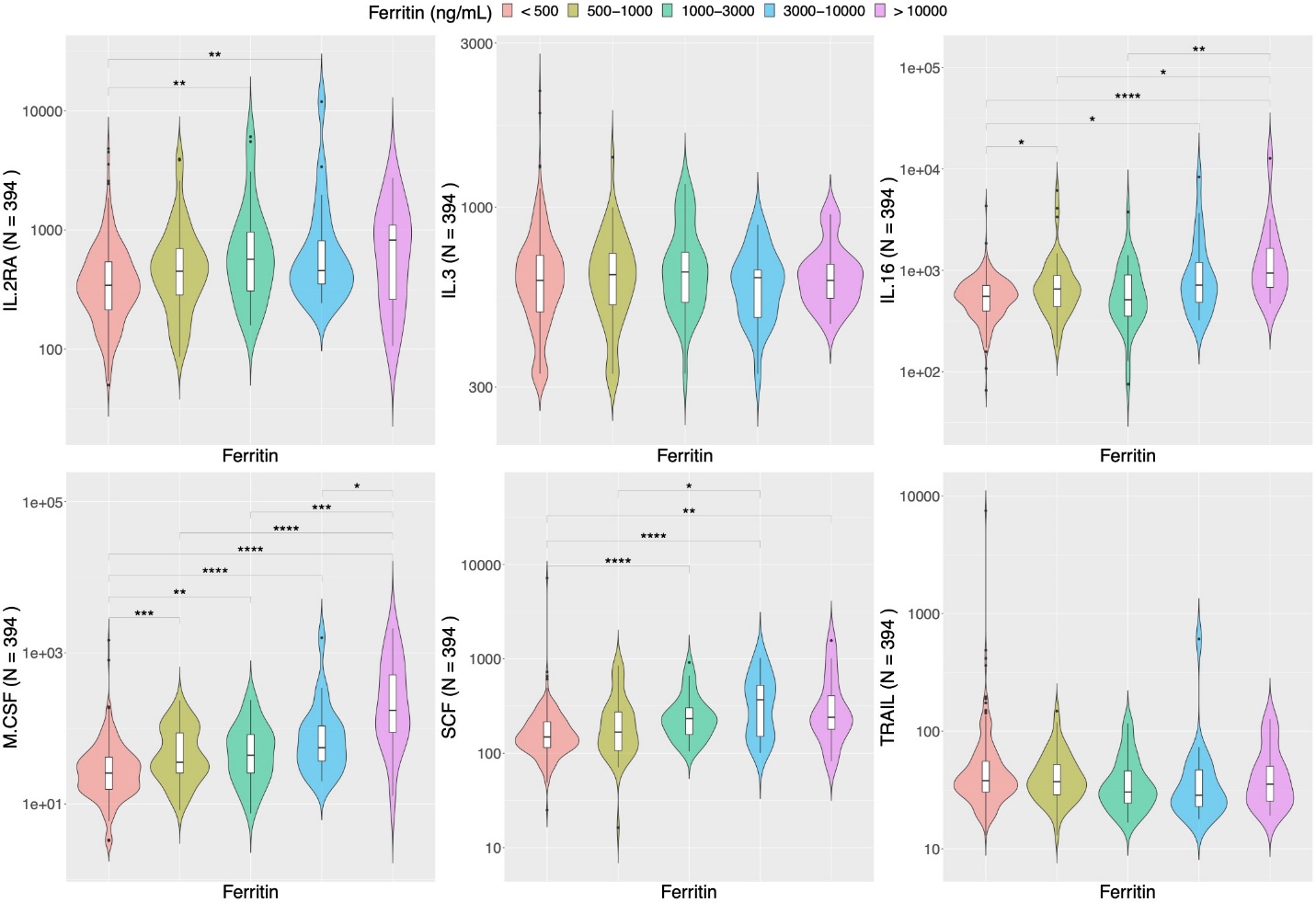


**eFigure 4. Cytokine value distribution by MAS**

**
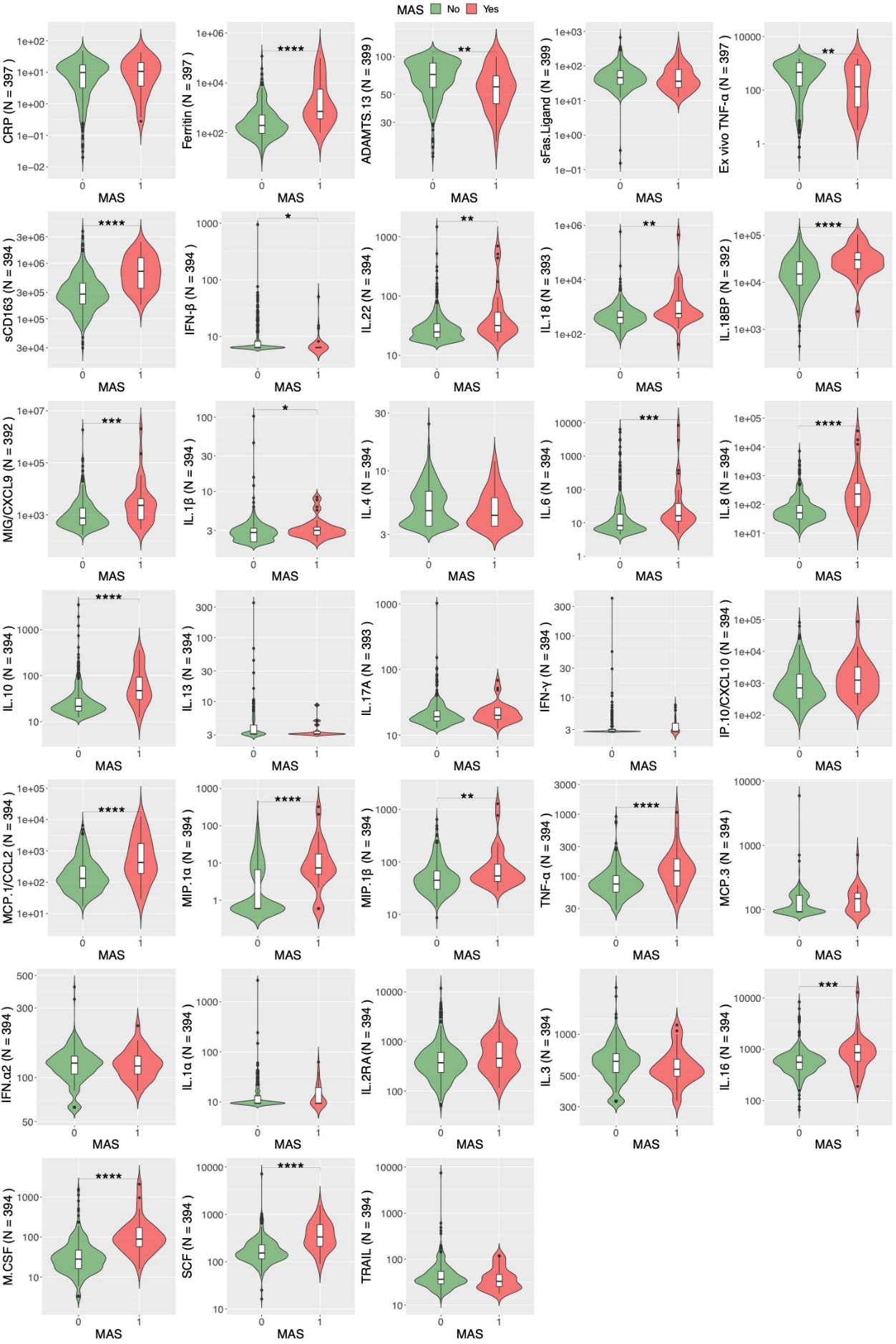
**

**eFigure 5. Whole blood ex vivo TNF response to endotoxin in children with and without pre-existing immunocompromised status**

**
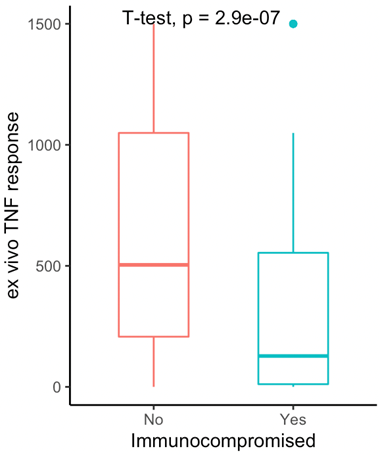
**

**eFigure 6. Whole blood ex vivo TNF response to endotoxin in children with bacterial infection or viral infection**


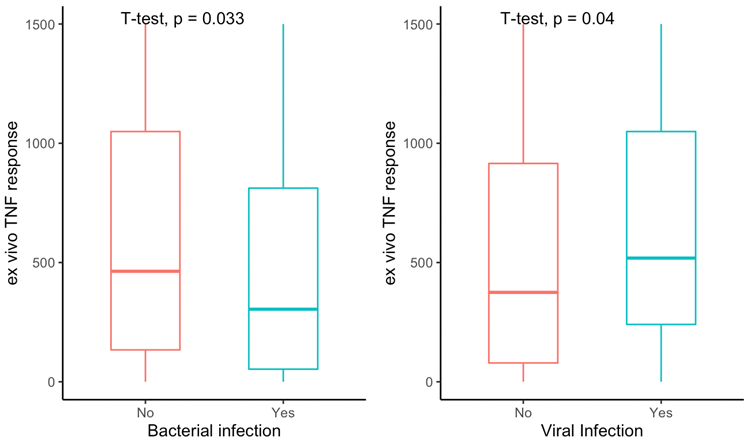

Supplement: Supplementary file 1 — Additional file 1. Supplemental Digital Content. [file 13054_2023_4628_MOESM1_ESM.docx]
